# Unfolding Immune Dysregulation in COPD: Identification of a Three-Gene Signature and Functional Validation of *TCF7* in Human Lung Tissue and T Lymphocytes

**DOI:** 10.3390/ijms27104231

**Published:** 2026-05-09

**Authors:** Zengrui Wang, Yue Yang, Le Wang, Zhuang Luo

**Affiliations:** 1 Department of Respiratory and Critical Care Medicine, The First Affiliated Hospital, Kunming Medical University, No. 1168, Chunrong West Road, Yuhua Sub-District, Chenggong District, Kunming 650500, China; 20240467@kmmu.edu.cn; 2 School of Clinical Oncology, Kunming Medical University, No. 1168, Chunrong West Road, Yuhua Sub-District, Chenggong District, Kunming 650500, China; 2024542084@kmmu.edu.cn (Y.Y.); 20242011@kmmu.edu.cn (L.W.)

**Keywords:** bioinformatics, COPD, gene signature, immune microenvironment, machine learning, single-cell analysis, regulatory mechanisms, in vitro pharmacological validation, T-cell homeostasis

## Abstract

Chronic obstructive pulmonary disease (COPD) is a leading global cause of mortality. Its molecular pathogenesis, especially systemic immune dysregulation, remains unclear. Public transcriptomic datasets underwent machine learning to identify biomarkers, which were validated in external cohorts and single-cell RNA-seq. In vitro and ex vivo validations included: qRT-PCR and ELISA in CSE/LPS-stimulated macrophages to assess drug efficacy (Celecoxib/Lovastatin); *RORC* overexpression; Western blotting in patient-derived primary T cells and *TCF7*-deficient Jurkat cells with genetic rescue; and immunofluorescence of human lungs. A three-gene signature (*RORC*, *TCF7*, and *CLEC4D*) consistently discriminated COPD. *CLEC4D* localized to myeloid cells, while *RORC*/*TCF7* mapped to lymphoid lineages. Celecoxib and Lovastatin attenuated macrophage inflammation, partially via *RORC* preservation. Crucially, the *TCF7* protein was depleted in COPD primary T cells. *TCF7* knockdown downregulated pro-caspase-8, which was fully reversed by *TCF7* re-expression. Immunofluorescence confirmed disrupted *TCF7*/caspase-8 spatial patterns in COPD lungs. This signature highlights innate hyperactivation and adaptive T-cell alterations in COPD, providing novel mechanistic insights into immune dysregulation and potential pharmacological targets.

## 1. Introduction

Chronic obstructive pulmonary disease (COPD) is a heterogeneous lung condition characterized by chronic respiratory symptoms due to abnormalities of the airways or alveoli that cause persistent, often progressive airflow obstruction [1,2]. It remains a leading cause of morbidity and mortality worldwide, posing a major public health challenge and substantial socioeconomic burden [3,4]. Despite advances in treatment and understanding of its clinical course, effective disease-modifying therapies remain limited, and the molecular mechanisms underlying COPD pathogenesis are still incompletely understood, partly because of its marked heterogeneity and multifactorial nature [5,6]. Early diagnosis and subtype-specific management, therefore, remain challenging, highlighting the need for reliable molecular biomarkers to support precision medicine in COPD [7].

Conventional diagnostic approaches, such as spirometry and imaging, provide important physiological and structural information but lack adequate sensitivity in detecting early or subclinical disease [8,9]. Recent technological advances in high-throughput sequencing, transcriptomics, and computational biology have enabled researchers to investigate COPD at the molecular level [10]. In particular, integrative bioinformatics and machine learning approaches have been increasingly applied to identify potential diagnostic markers, prognostic genes, and therapeutic targets [11,12].

Several studies have employed differential gene expression (DEG) and network-based analyses—such as weighted gene co-expression network analysis (WGCNA)—in conjunction with machine learning algorithms including Boruta and LASSO regression to identify hub genes and construct predictive diagnostic models [13,14]. Moreover, single-cell RNA sequencing (scRNA-seq) has provided novel insights into the immune and cellular microenvironment of COPD, revealing the complex interplay among epithelial, endothelial, and immune cells [15,16]. Integrating multi-omics data with immune profiling has further deepened our understanding of inflammation, oxidative stress, and mitochondrial dysfunction in COPD [17].

However, most existing studies focus on limited datasets or single-omics analyses, often lacking comprehensive integration with immune and regulatory networks [18]. The biological significance and immunological roles of identified biomarkers often remain insufficiently validated. Therefore, a systematic, multi-level integration combining bulk transcriptomics, machine learning, immune infiltration analysis, and single-cell data is needed to identify robust COPD biomarkers and explore their regulatory mechanisms.

In this study, we integrated public datasets using machine learning (Boruta and LASSO) to identify diagnostic biomarkers for COPD. We explored their immunoregulatory roles and cellular localizations via regulatory networks, immune infiltration, and single-cell RNA sequencing. Moving beyond in silico predictions like molecular docking, we established biological causality through rigorous functional validations. Specifically, we evaluated candidate drugs in macrophage inflammatory models and utilized patient-derived primary T cells alongside genetic rescue to demonstrate the pathological downregulation of the *TCF7*–caspase-8 axis. Finally, in situ structural alterations and biomarker expressions were validated using clinical lung tissues via hematoxylin and eosin (H&E) and immunofluorescence staining.

## 2. Results

### 2.1. Overview of the Study Design

**Figure 1:**
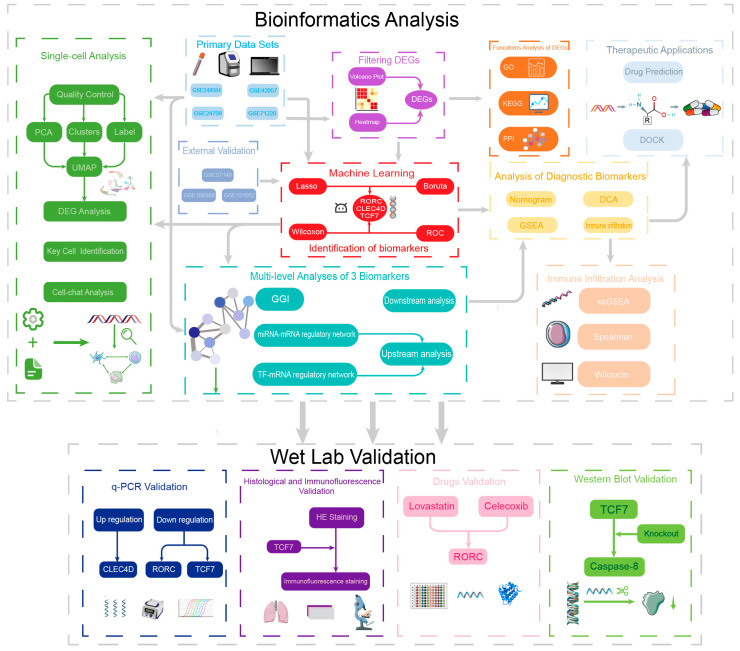
Flowchart of the study.

To elucidate systemic immune dysregulation in COPD and identify mechanistically relevant biomarkers, we implemented a multi-stage pipeline. First, large-scale blood transcriptomic data were analyzed for differential expression, followed by machine learning (Boruta/LASSO) to establish a diagnostic gene signature validated across multiple cohorts. Next, in silico profiling (immune infiltration, single-cell RNA-seq, and molecular docking) was performed. Finally, computational predictions were experimentally validated using qRT-PCR, ELISA, patient-derived primary T lymphocytes, target overexpression/genetic rescue models, and immunofluorescence in human lung tissues. The workflow is illustrated in Figure 1.

### 2.2. Identification of Differentially Expressed Genes (DEGs)

First, we established the clinically relevant baseline characteristics of the training cohort (GSE71220). After rigorously excluding patients utilizing statins to avoid pharmacological confounding, the final analysis cohort comprised 405 patients with COPD and 44 healthy controls (Table 1). As expected for this disease, the patients with COPD exhibited significantly lower lung function indices (FEV1% predicted and FEV1/FVC, *p* < 0.001) and a higher prevalence of smoking compared to controls. Notably, the sex distribution was well-balanced between groups (*p* = 1).

**Figure 2:**
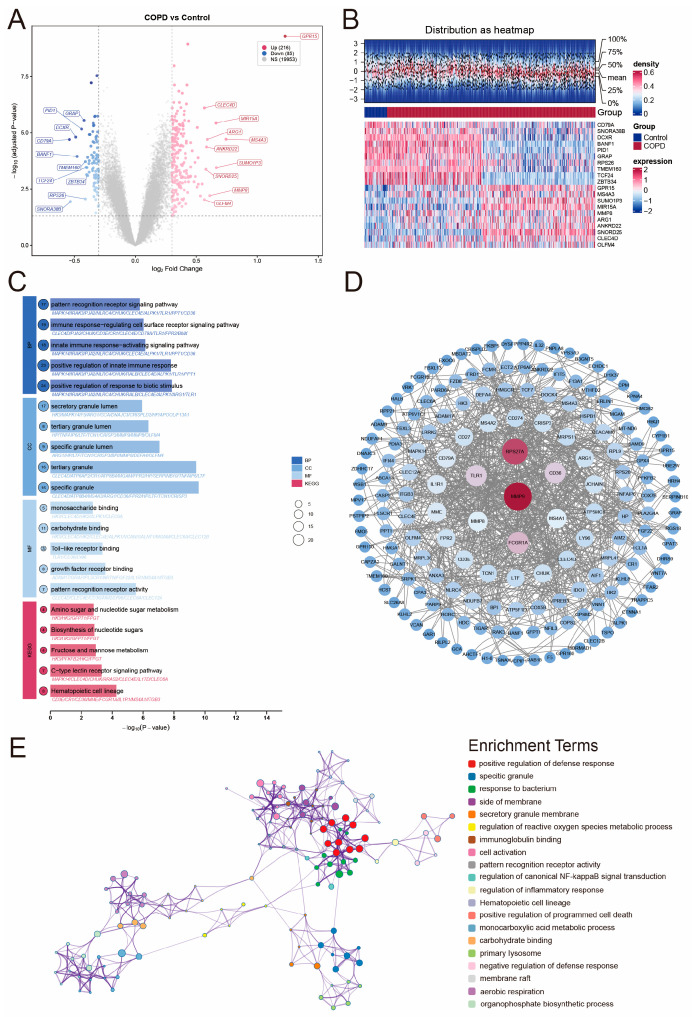
Identification of differentially expressed genes and functional enrichment analysis: (**A**) Volcano plot of differentially expressed genes in the GSE71220 training cohort. Significantly upregulated genes (log_2_FC > 0.3, Padj < 0.05) are shown in orange, and significantly downregulated genes (log_2_FC <−0.3, Padj < 0.05) in blue. The top ten upregulated and downregulated genes are labeled. (**B**) Heatmap of the top 20 DEGs with sample density distribution. The upper panel shows the density distribution of all the DEGs, indicating stable expression patterns across the samples. The lower panel displays the heatmap of the top 20 DEGs, with blue representing the control samples and red representing the COPD samples. Notably, distinct internal sub−clusters were observed within the COPD group, reflecting the clinical heterogeneity of the cohort. (**C**) Gene Ontology and KEGG enrichment analysis of DEGs. Enrichment terms are categorized into biological process, cellular component, molecular function, and KEGG pathways. The x-axis represents the -log_10_-adjusted *p*-value, and the number of genes enriched in each term is indicated by the number inside the circles on the left side of each bar. (**D**) Protein–protein interaction network of DEGs. Nodes represent proteins, and edges indicate experimentally validated or predicted interactions. Hub genes with the highest degree centrality, including *MMP9*, *RPS27A*, *FCGR1A*, *CD36*, and *TLR1*, are centrally positioned. (**E**) Network of enriched GO and KEGG terms. Nodes represent individual enrichment terms, and edges indicate shared genes between terms. Different colors represent distinct functional categories. Nearly all the terms were closely interconnected except for the term “regulation of reactive oxygen species metabolic process”, which was relatively isolated.

Subsequently, we identified differentially expressed genes between the patients with COPD and healthy controls using the GSE71220 dataset. Applying the thresholds |log_2_FC| > 0.3 and Benjamini–Hochberg-adjusted *p*-value (Padj) < 0.05, a total of 301 DEGs were identified, of which 216 were upregulated and 85 were downregulated. The volcano plot (Figure 2A) illustrates the distribution of these genes, with upregulated genes (orange) such as *CLEC4D*, *MIR15A*, and *GPR15*, and downregulated genes (blue) such as *CD79A*, *DCXR*, and GRAP. Highly differentially expressed genes are marked via labels. The heatmap (Figure 2B) displays the expression patterns of these DEGs across the samples, showing clear segregation between the COPD and control groups. Notably, we observed distinct internal sub-clusters within the COPD group in the heatmap. This internal heterogeneity likely reflects the broad clinical spectrum of the GSE71220 cohort (encompassing varying GOLD stages and smoking histories), a potential confounding variance that we subsequently addressed through covariate-adjusted sensitivity analysis.

### 2.3. Functional Enrichment and Pathway Analysis

To elucidate the biological functions and signaling pathways associated with the identified DEGs, we performed GO and KEGG enrichment analyses. The analysis revealed that the DEGs were predominantly enriched in immune-related processes and metabolic activities (Figure 2C).

In the Gene Ontology (GO) analysis, biological process (BP) terms were significantly focused on the activation of the innate immune system. The top enriched terms included “positive regulation of response to biotic stimulus”, “positive regulation of innate immune response”, and “innate immune response-activating signaling pathway”. Consistent with these biological processes, molecular function (MF) analysis highlighted receptor activities essential for pathogen detection. Key enriched terms included “pattern recognition receptor activity”, “Toll-like receptor binding”, and “carbohydrate binding”. Cellular component (CC) analysis provided insight into the cellular source of these signatures. The DEGs were significantly localized to secretory granule structures, such as “specific granule”, “tertiary granule”, and “secretory granule lumen”. Focusing on KEGG pathway analysis further corroborated these findings. The most significant pathways included “Hematopoietic cell lineage” and the “C-type lectin receptor signaling pathway”, which align with the immune activation observed in the GO analysis. Additionally, metabolic pathways such as “Fructose and mannose metabolism” and “Amino sugar and nucleotide sugar metabolism” were enriched.

The network of enrichment terms (Figure 2E) shows that nearly all the terms were closely interconnected, with the exception of the “regulation of reactive oxygen species metabolic process” term, which appeared relatively isolated.

To explore the interaction of proteins, we used the STRING database and Cytoscape to construct a protein–protein interaction (PPI) network (Figure 2D). The network confirmed that these genes interact in a tightly regulated system, with hub genes such as *MMP9*, *RPS27A*, *FCGR1A*, *CD36*, and *TLR1* forming central nodes involved in the immune response.

### 2.4. Biomarker Selection Using Machine Learning

To identify robust biomarkers for COPD, we employed machine learning algorithms in the training cohort GSE71220, specifically Boruta and LASSO regression. Both methods helped narrow down the candidate biomarkers with the intersection of two machine learning results being the hub genes for the final biomarkers.

**Figure 3:**
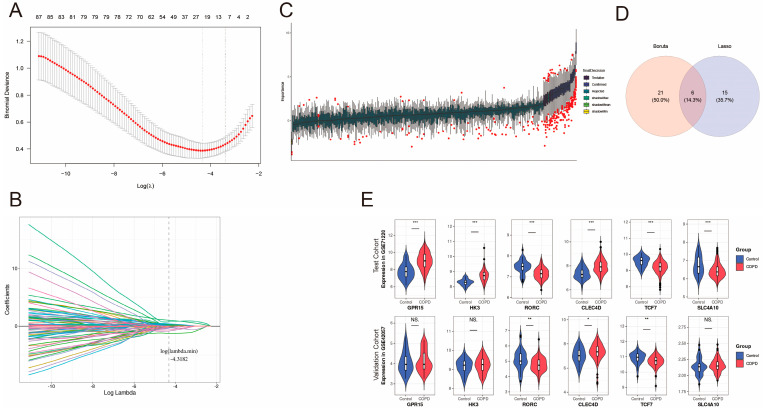
Machine learning-based biomarker selection: (**A**) LASSO regression curve for feature selection in the training cohort GSE71220. The x-axis shows the log of the regularization parameter λ, and the y-axis shows the partial likelihood deviance. Two vertical dashed lines indicate the optimal λ values: the left dashed line corresponds to λ.min (the value that minimizes the cross-validated error), and the right dashed line corresponds to λ.1se (the largest λ within one standard error of the minimum). The optimal penalty parameter λ = 0.013 (λ.min) was selected, yielding 21 non-zero coefficient genes including *HK3*, *GPR15*, and *RORC*. (**B**) LASSO coefficient paths. Each colored line represents the trajectory of a gene coefficient as the regularization penalty increases (decreasing log λ). The vertical dashed line indicates the selected λ.min at log λ = −4.3182, corresponding to the optimal model complexity. (**C**) Boruta feature importance assessment. Boxplots display the importance scores of each feature across iterative random forest runs. The y-axis represents the importance score; the vertical position (median) of each boxplot reflects the feature’s importance, while the box length represents the interquartile range, indicating variability across runs. Core predictors (green) are distinguished from tentative (blue) and rejected (red) features. (**D**) Venn diagram showing the overlap between genes selected by LASSO and Boruta. Six hub genes (*GPR15*, *HK3*, *RORC*, *CLEC4D*, *TCF7*, and *SLC4A10*) were identified by both algorithms and retained for further analysis. (**E**) Validation of biomarker expression in the training and validation cohorts. Violin plots display the expression levels of the six hub genes. The upper panels show expression in the training cohort GSE71220, and the lower panels show expression in the internal validation cohort GSE42057 significance: * *p* < 0.05, ** *p* < 0.01, *** *p* < 0.001.

The LASSO regression curve illustrates the relationship between log(λ) (regularization parameter) and the coefficients of the genes in Figure 3A,B. We then decide the optimal penalty parameter (λ = 0.013) under which 21 genes like *HK3*, *GPR15*, and *RORC*. The Boruta algorithm (Figure 3C) identified 27 relevant genes while presenting their feature importance scores. In this visualization, features are arrayed along the x-axis, and their importance scores are plotted on the y-axis as a series of boxplots. The overall importance of each feature is indicated by its vertical position (median score), whereas the length of the box represents the interquartile range (IQR), indicating the variability of the importance score across the iterative runs. There were six genes (Figure 3D) collected as the hub biomarkers selected by both methods, highlighting their robustness. Those genes are *GPR15*, *HK3*, *RORC*, *CLEC4D*, *TCF7* and *SLC4A10*. To further filter those hub genes, we employed the expression test while testing its significance. Figure 3E shows 12 boxplots comparing the expression of six biomarkers in both the training dataset and the testing dataset (GSE42057). The plot reveals that *RORC* and *TCF7* are significantly downregulated in the COPD samples, while *CLEC4D* is significantly upregulated, confirming their differential expression and robustness for further analysis.

### 2.5. Diagnostic Performance and Clinical Translation Model

**Figure 4:**
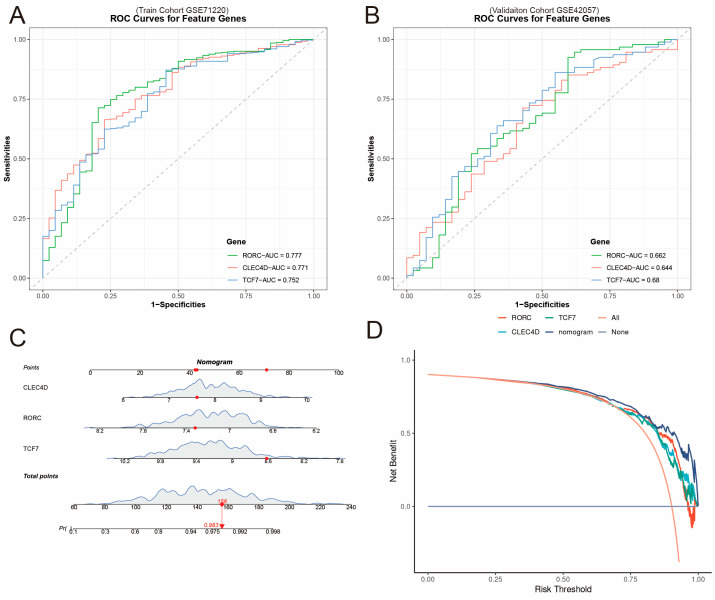
Validation of the diagnostic performance of the three-gene signature: (**A**) Receiver operating characteristic curves for the three biomarkers in the training cohort GSE71220. The area under the curve values are 0.777 for *RORC*, 0.771 for *CLEC4D*, and 0.752 for *TCF7*. *RORC* is shown as a green stepped line, *CLEC4D* in red, and *TCF7* in blue. (**B**) Receiver operating characteristic curves in the internal validation cohort GSE42057. The area under the curve values are 0.662 for *RORC*, 0.644 for *CLEC4D*, and 0.680 for *TCF7*. Color coding follows the same scheme as in panel A. (**C**) Nomogram for predicting COPD probability based on the expression levels of the three-gene signature. Each gene contributes a score corresponding to its expression value; the total score is summed to estimate the predicted probability. As an example, a randomly selected patient with a total score of 156 corresponds to a predicted probability of 0.983. (**D**) Decision curve analysis evaluating the clinical utility of the three-gene signature. The curves represent the net benefit of clinical decisions based on *RORC*, *TCF7*, and *CLEC4D*, and the combined nomogram across a range of threshold probabilities.

We systematically evaluated the diagnostic performance of the three-gene (*RORC*, *TCF7*, *CLEC4D*) signature. In the training set, the ROC curve analysis (Figure 4A) showed AUC values ranging from 0.752 to 0.777 for individual genes, indicating good discriminatory ability. In the independent validation set (GSE42057), the signature also demonstrated stable diagnostic performance (Figure 4B).

To facilitate clinical application, we constructed a nomogram incorporating the expression levels of these three genes (Figure 4C). The calibration curves showed good agreement between the predicted and observed probabilities (Figure S1). The decision curve analysis (DCA, Figure 4D) indicated that using this three-gene signature for clinical decisions provided a higher net benefit across a wide range of threshold probabilities compared to “treat all” or “treat none” strategies, demonstrating its potential clinical utility with all the genes and the gene model all higher than “Treat all.”

### 2.6. Cross-Tissue and External Validation of Three Biomarkers

To rigorously evaluate the robustness and clinical generalizability of the three-gene signature, we validated its expression patterns in three independent external datasets comprising both peripheral blood (GSE56766 and GSE306950) and lung tissue (GSE106986).

**Figure 5:**
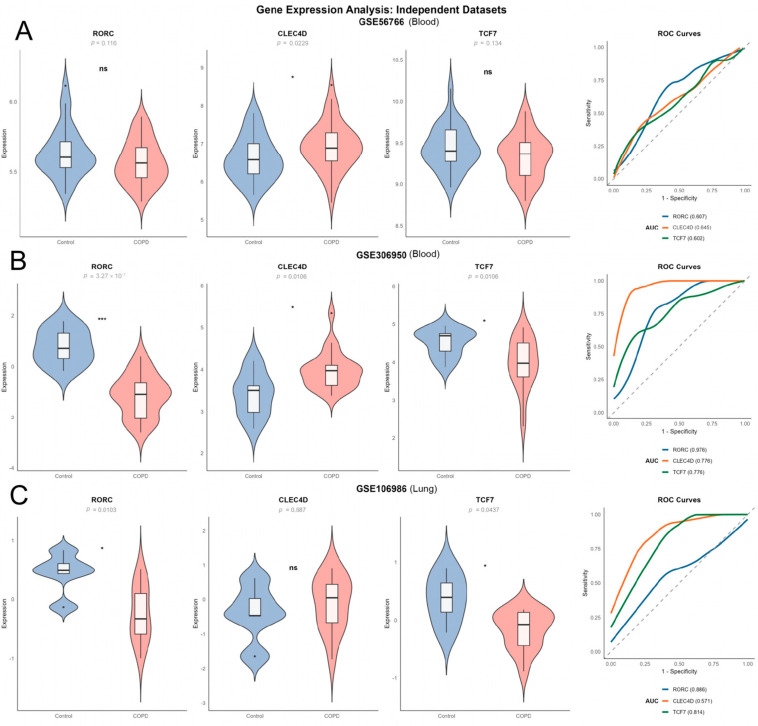
External validation of the three-gene signature across independent cohorts: (**A**) Validation in the peripheral blood cohort GSE56766. The violin plots show the expression levels of the three biomarkers in patients with COPD and healthy controls. *CLEC4D* was significantly upregulated, while *RORC* and *TCF7* did not show statistically significant differences. The receiver operating characteristic curves for each biomarker are shown to the right, with the area under the curve values ranging from 0.602 to 0.654. *RORC* is depicted in blue, *CLEC4D* in orange, and *TCF7* in green. (**B**) Validation in the peripheral blood cohort GSE306950. The violin plots show that *RORC* and *TCF7* were significantly downregulated, whereas *CLEC4D* was significantly upregulated in the COPD samples compared to controls. The corresponding ROC curves yielded area under the curve values of 0.976 for *RORC*, 0.776 for *CLEC4D*, and 0.776 for *TCF7*. Color coding follows the same scheme as in panel (**A**). (**C**) Validation in the lung tissue cohort GSE106986. The violin plots show that both *RORC* and *TCF7* were significantly downregulated in COPD lung tissue, while *CLEC4D* did not reach statistical significance. The receiver operating characteristic analysis revealed area under the curve values of 0.886 for *RORC*, 0.571 for *CLEC4D*, and 0.814 for *TCF7*. Color coding is consistent with previous panels. Significance: * *p* < 0.05, *** *p* < 0.001.

Consistent with the findings in the training and internal validation sets, the three genes displayed concordant expression trends across the external blood and lung cohorts (Figure 5A–C). Notably, the model exhibited exceptional diagnostic performance in the GSE306950 dataset. All three biomarkers were significantly dysregulated, with *RORC* achieving a remarkable diagnostic accuracy (AUC = 0.976), while *TCF7* and *CLEC4D* also demonstrated strong predictive power (both AUC = 0.776). This confirms that the identified signature is highly stable and reproducible in peripheral circulation across different populations.

To determine whether these systemic biomarkers reflect local pulmonary pathology, we examined their expression in lung tissue samples (GSE106986). While the expression trends remained consistent, *RORC* and *TCF7* emerged as the most robust candidates in the target organ. Both genes maintained statistical significance (*p* < 0.05) in lung tissue, with the diagnostic AUC values exceeding 0.80 (Figure 5C). This dual-compartment validation—showing high diagnostic value in both peripheral blood and lung tissue—suggests that *RORC* and *TCF7* are not merely byproducts of the blood environment of COPD but are intrinsically linked to the core pathological mechanisms within the lung.

Consequently, given their superior performance in identifying COPD pathology at both the systemic and tissue levels, *RORC* and *TCF7* were prioritized for further mechanistic exploration and experimental validation.

### 2.7. Gene Set Enrichment and Regulatory Mechanisms

**Figure 6:**
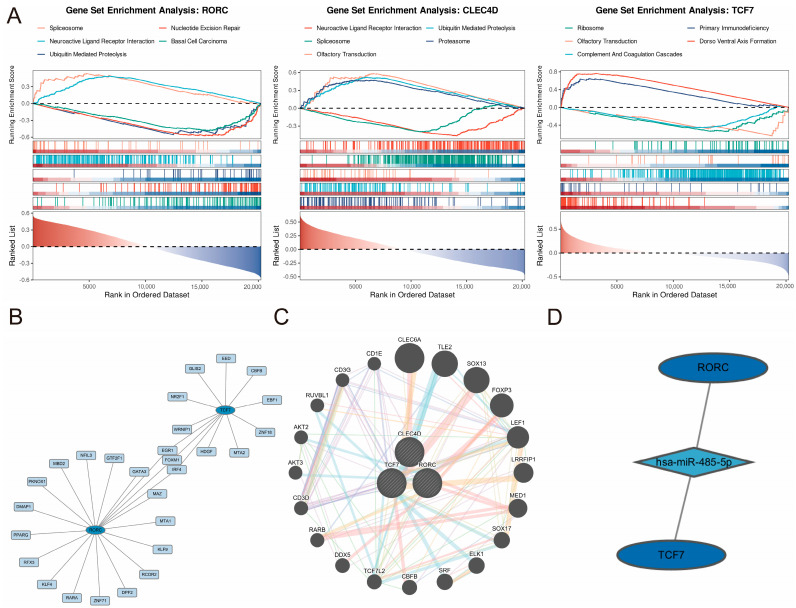
Gene set enrichment analysis and regulatory network construction: (**A**) Gene set enrichment analysis of the three biomarkers. The GSEA enrichment plots display the top five significantly enriched pathways for *RORC*, *CLEC4D*, and *TCF7*. Positive enrichment for *RORC* in neuroactive ligand–receptor interaction (NES = 3.05) and negative enrichment in spliceosome and ubiquitin−mediated proteolysis pathways (NES < −2.7) are shown. For *CLEC4D*, positive enrichment in spliceosome, ubiquitin−mediated proteolysis, and proteasome pathways, and negative enrichment in neuroactive ligand–receptor interaction and olfactory transduction are displayed. For *TCF7*, positive enrichment in the ribosome and primary immunodeficiency pathways (NES = 2.14) and negative enrichment in the complement and coagulation cascades are presented. (**B**) Transcription factor−miRNA−mRNA regulatory network for *TCF7* and *RORC*. The network was constructed using the GSE24709 dataset and visualized with Cytoscape. The nodes represent transcription factors (triangles), miRNAs (diamonds), and target genes (circles). *IRF4*, *FOXM1*, and *EGR1* are shown as transcription factors connecting *TCF7* and *RORC*. The network is presented in a blue color theme. (**C**) Gene–gene interaction network for *TCF7*, *CLEC4D*, and *RORC*. Interactions were retrieved from GeneMANIA, integrating evidence from multiple public databases and published studies. Edge colors represent seven interaction types: physical interactions (77.64%), co−expression (8.01%), predicted interactions (5.37%), co−localization (3.63%), genetic interactions (2.87%), pathway (1.88%), and shared protein domains (0.60%). (**D**) Transcription factor−miRNA−mRNA regulatory network highlighting miRNA connections. hsa−miR−485−5p is indicated as a key miRNA connecting *TCF7* and *RORC*. The network follows the same blue color theme as panel (**B**).

To elucidate the underlying biological functions and signaling pathways driven by the three identified biomarkers, Gene Set Enrichment Analysis (GSEA) was conducted (Figure 6A). The analysis revealed distinct regulatory patterns for each gene. *RORC* exhibited significant positive enrichment in neuroactive ligand–receptor interactions (NES = 3.05), while essential cellular processes such as the spliceosome and ubiquitin-mediated proteolysis were markedly suppressed (NES < −2.7). In contrast, *CLEC4D* displayed an inverse enrichment profile, showing strong activation of the spliceosome, ubiquitin-mediated proteolysis, and proteasome pathways, coupled with a downregulation of neuroactive ligand–receptor interactions and olfactory transduction. *TCF7* was primarily associated with translational and immune-related machinery, showing significant enrichment in the ribosome and primary immunodeficiency pathways (NES = 2.14), whereas the complement and coagulation cascades were negatively enriched. These findings suggest that while *RORC* and *CLEC4D* may function through opposing mechanisms in protein processing and signal transduction, *TCF7* is specifically implicated in ribosomal function and immune deficiency-related signaling.

To further analyze the regulation and explore the connection of the three biomarkers, transcription factor (TF)-miRNA-mRNA regulatory networks were constructed (Figure 6B) using GSE24709, which revealed complex interactions between *TCF7* and *RORC*. *IRF4*, *FOXM1* and *EGR1* are genes that connect *TCF7* and *RORC*. Furthermore, we found that hsa-miR-485-5p was identified as a key miRNA connecting *TCF7* with *RORC* in Figure 6D.

The gene–gene interaction (GGI) analysis for *TCF7*, *CLEC4D*, and *RORC* integrates evidence from multiple public databases and published studies, categorizing interactions into seven major types in Figure 6C. The overall distribution of interaction evidence types is summarized below, with physical interactions accounting for the majority (77.64%), followed by co-expression (8.01%), predicted interactions (5.37%), co-localization (3.63%), genetic interactions (2.87%), pathway (1.88%), and shared protein domains (0.60%). Detailed contributions from individual studies and datasets are presented in the following sections. Detailed information is conserved in Supplementary Materials.

### 2.8. Immune Cell Infiltration and Correlation Analysis

**Figure 7:**
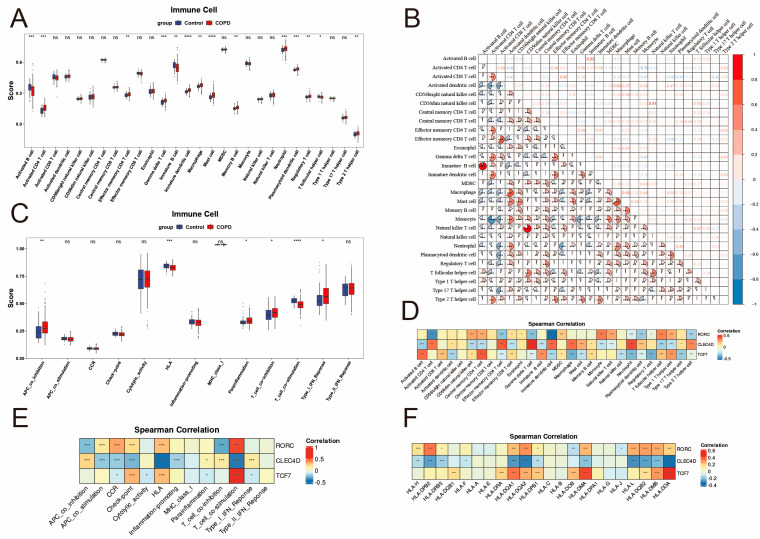
Immune landscape and biomarker associations: (**A**) Heatmap of ssGSEA-based quantification for 28 immune cell types in patients with COPD versus healthy controls. Red indicates higher relative abundance, and blue indicates lower abundance. (**B**) Correlation heatmap of differentially abundant immune cell subpopulations. Spearman correlations are represented by color intensity (red: positive; blue: negative). (**C**) Heatmap of immune−related functional pathway enrichment scores in COPD versus controls. (**D**–**F**) Correlation heatmaps illustrating the associations between the three diagnostic biomarkers (*CLEC4D*, *RORC*, and *TCF7*) and (**D**) immune cell subpopulations, (**E**) functional pathways, and (**F**) HLA gene expression levels. Significance: * *p* < 0.05, ** *p* < 0.01, *** *p* < 0.001. Statistical significance is denoted as follows: ****, *p* < 0.0001.

To systematically evaluate the immunological heterogeneity between patients with COPD and healthy controls, we quantified the relative abundance of 28 immune cell types using the ssGSEA algorithm. The analysis revealed a widespread remodeling of the immune microenvironment in the COPD group (Figure 7A).

In the innate immune compartment, we observed a broad upregulation of myeloid-derived cells and granulocytes. Specifically, Immature dendritic cells and Mast cells showed the most substantial increase (*p* < 0.001), followed by significantly elevated levels of neutrophils, macrophages, and plasmacytoid dendritic cells (*p* < 0.01). Regarding adaptive immunity, the T-cell landscape was characterized by significant activation and specific lineage skews. The patients with COPD exhibited higher infiltration of Activated CD4 T cells, Gamma delta (γδ) T cells, and Effector memory CD4 T cells. Notably, both Regulatory T cells (Tregs) and Type 2 T helper (Th2) cells were upregulated, whereas T follicular helper (Tfh) cells were significantly downregulated. The B cell compartment displayed a divergent pattern: while Memory B cells were increased, the levels of Activated B cells and Immature B cells were significantly lower in the COPD group compared to controls.

To explore the intricate relationships among these differentially abundant immune populations, we performed a correlation analysis. As shown in Figure 7B, the strongest positive correlation was observed between Immature B cells and Activated B cells (r = 0.93, *p* < 0.001), indicating a close developmental link. Within the innate compartment, CD56dim natural killer cells exhibited a near-perfect correlation with natural killer T cells (r = 0.81, *p* < 0.001). The T-cell compartment revealed distinct clusters: Activated CD4 T cells were strongly correlated with Effector memory CD4 T cells (r = 0.46, *p* < 0.001) and γδ T cells (r = 0.59, *p* < 0.001), suggesting coordinated activation of these subsets. Notably, monocytes displayed a strong negative correlation with Activated CD4 T cells (r = −0.79, *p* < 0.001), highlighting a potential antagonistic relationship between myeloid and adaptive compartments in the COPD immune microenvironment.

To further characterize the functional immune landscape, we evaluated 13 molecular pathways, revealing a distinct pattern of dysregulation marked by suppressed activation signals and heightened inhibitory mechanisms (Figure 7C). Specifically, antigen presentation and priming capacities were impaired, evidenced by the significant downregulation of HLA and T-cell co-stimulation (*p* < 0.001), while immunosuppressive pathways such as APC and T-cell co-inhibition were concurrently upregulated (*p* < 0.05), aligning with the observed Treg expansion. Despite this suppressive environment, a persistent inflammatory state was indicated by the upregulation of Parainflammation and Type I IFN response (*p* < 0.05).

Given the extensive immune remodeling observed in COPD, we next investigated the specific associations between the three diagnostic biomarkers (*CLEC4D*, *RORC*, and *TCF7*) and the immune cell subpopulations, functional pathways, and HLA gene expression levels (Figure 7D–F).

*CLEC4D* showed moderate positive correlations with innate immune cells, specifically neutrophils (r = 0.44), macrophages (r = 0.37), and γδ T cells (r = 0.49), while exhibiting negative correlations with Activated B cells and T follicular helper cells. In terms of immune functions, *CLEC4D* was negatively correlated with T-cell co-stimulation (r = −0.39) and the HLA pathway (r = −0.41), but positively associated with Parainflammation and Type I IFN response. Consistently, at the gene level, *CLEC4D* showed significant negative correlations with multiple MHC Class II molecules, including *HLA-DOA*, *HLA-DQA2*, and *HLA-L*.

*RORC* presented a different correlation pattern, showing negative correlations with Immature dendritic cells (r = −0.52) and Activated CD4 T cells (r = −0.45), but positive associations with monocytes (r = 0.35) and T follicular helper cells (r = 0.33). Functionally, *RORC* was positively correlated with T-cell co-stimulation (r = 0.53) and the CCR pathway, while negatively correlated with T-cell co-inhibition. Regarding specific HLA genes, *RORC* showed positive associations with *HLA-DPB2*, *HLA-DQA2*, and *HLA-DQB2*.

*TCF7* exhibited significant positive correlations with Central memory CD4 T cells (r = 0.41) and Activated B cells (r = 0.37), whereas it was negatively correlated with Regulatory T cells (r = −0.37), macrophages, and neutrophils. Among the functional pathways, *TCF7* demonstrated the strongest positive correlation with T-cell co-stimulation (r = 0.57) and the HLA pathway. This association was further reflected at the molecular level, where *TCF7* was positively correlated with key MHC Class II genes, particularly *HLA-DOA*, *HLA-DMA*, and *HLA-DQA1* (*p* < 0.001).

### 2.9. Single-Cell RNA Sequencing and CellChat Analysis

**Figure 8:**
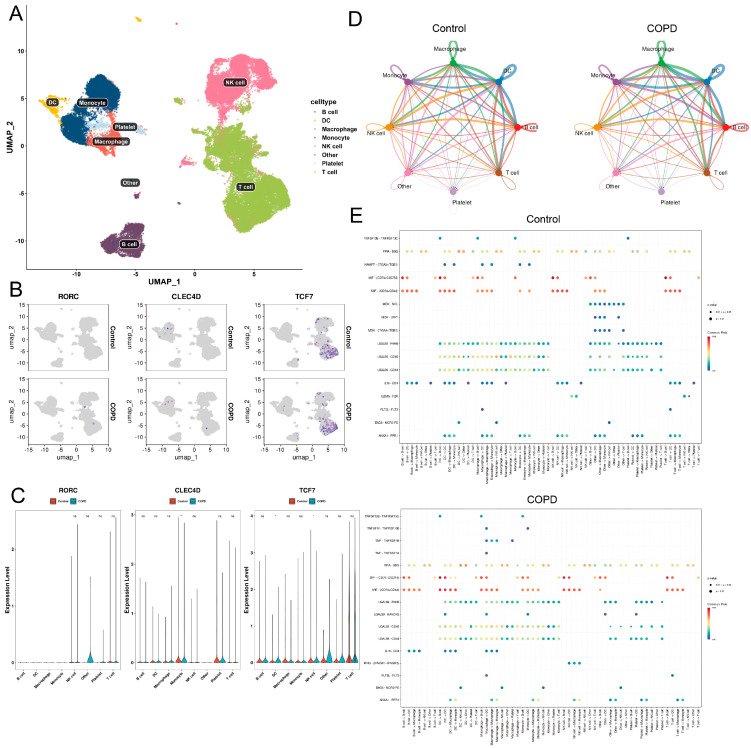
Single-cell transcriptomic landscape and intercellular communication analysis: (**A**) UMAP visualization of the GSE249584 single-cell RNA-seq dataset. The cells were clustered into eight distinct immune cell types based on marker gene expression. (**B**) UMAP plots showing the expression distribution of *CLEC4D*, *RORC*, and *TCF7* in the control and COPD samples. *CLEC4D* was predominantly expressed in myeloid populations, whereas *RORC* and *TCF7* were largely restricted to lymphoid lineages. (**C**) Violin plots comparing the expression levels of the three biomarkers between control and COPD groups across different cell types. *CLEC4D* showed upregulation in monocytes, macrophages, and dendritic cells in the COPD samples. *RORC* expression was downregulated in T cells and natural killer cells, whereas *TCF7* exhibited elevated expression in natural killer and B cell clusters in COPD compared to controls. * *p* < 0.05, ** *p* < 0.01. (**D**) CellChat network analysis showing the overall communication strength and weight among immune cell populations in control and COPD conditions. Edge thickness represents the probability of intercellular communication. (**E**) Bubble plot displaying significant ligand–receptor pairs between cell populations in control and COPD groups. Circle size indicates communication probability, and color intensity represents relative expression levels.

To determine the cellular localization of the identified biomarkers, we analyzed scRNA-seq data (GSE249584), revealing distinct immune clusters via UMAP (Figure 8A). Expression analysis (Figure 8B,C) confirmed lineage-specific patterns: *CLEC4D* was predominantly expressed in the myeloid compartment, specifically enriched in monocytes, macrophages, and DCs, with notable upregulation in the COPD group. In contrast, *RORC* and *TCF7* were restricted to the lymphoid lineage. *RORC* expression was downregulated in T and NK cells in the COPD samples, while *TCF7*, though constitutively expressed in T cells, showed elevated expression levels in the NK and B cell clusters of the patients with COPD compared to the controls.

Intercellular communication analysis via CellChat (Figure 8D,E) indicated a global reduction in network complexity in COPD, with the total number of interactions decreasing from 261 to 218. This reduction was characterized by a loss of homeostatic signaling pathways, including Midkine (MK), Pleiotrophin (PTN), and Galectin (GALECTIN). Structurally, monocytes and macrophages maintained high signaling probability as dominant sources of outgoing signals, whereas adaptive immune crosstalk, particularly the communication between B cells and T cells, was notably impaired.

The Macrophage Migration Inhibitory Factor (MIF) pathway constituted the most predominant communication axis in the COPD microenvironment. High-probability interactions were observed involving MIF binding to CD74 + CXCR4 and CD74 + CD44 receptor complexes. While present in controls, the COPD network displayed a consolidation of these interactions, with dendritic cells, NK cells, and T cells acting as prominent sources targeting B cells and monocytes. This amplification suggests a reinforced connectivity between innate immune effectors and the B cell compartment in the disease state.

A distinctive feature of the COPD landscape was the de novo emergence of specific pro-inflammatory signaling pairs absent in the top-ranked control interactions. Type II Interferon (IFN-II) signaling was identified, characterized by IFNG originating from NK cells and targeting myeloid populations (macrophages, monocytes, and DCs) via the IFNGR1 + IFNGR2 complex. Concomitantly, the TNF signaling pathway was activated specifically through autocrine TNF-TNFRSF1B interactions within the macrophage population, indicating a shift toward a Th1-type inflammatory milieu driven by NK–myeloid crosstalk.

Finally, the Galectin signaling pathway exhibited a critical shift in receptor usage. While homeostatic interactions involving LGALS9 (Galectin-9) binding to PTPRC (CD45) and CD44 were preserved in both groups, the COPD group uniquely exhibited the appearance of the LGALS9-HAVCR2 (TIM-3) axis, primarily driven by macrophages and DCs acting on other myeloid cells. Additionally, the IL16 signaling pathway (IL16-CD4) was observed connecting macrophages to CD4+ targets. This specific emergence of the inhibitory TIM-3 receptor axis, contrasted with the disappearance of homeostatic MDK and PTN pathways, highlights a complex remodeling of immunoregulatory networks in COPD.

### 2.10. Molecular Docking and Drug Prediction

To identify COPD therapeutic candidates, we integrated transcriptomic reversal predictions (cMap) with drug–target networks (DSigDB). As shown in Table 2, Digoxin, a known RORγt antagonist, was successfully retrieved, validating the screening approach. Lovastatin and Celecoxib emerged as promising repositioning candidates capable of targeting *RORC* and reversing the COPD signature. Detailed information is collected in Table S2.

**Figure 9:**
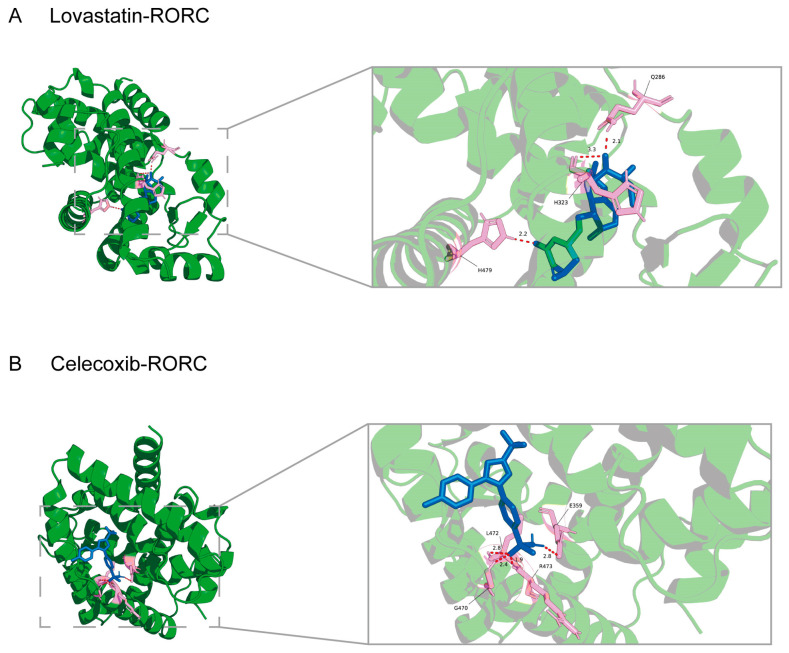
Molecular docking analysis of *RORC* with candidate drugs: (**A**) Three−dimensional structure of *RORC* (green) in complex with Lovastatin (blue). The protein is depicted as a green ribbon diagram, with the ligand shown in blue. The residues involved in ligand binding are highlighted in pink, and hydrogen bonds are represented by red dashed lines. Interacting residues include His479 (hydrogen bond distance 2.2 Å), His323 (3.3 Å), and Gln286 (2.1 Å). The binding affinity is −9.0 kcal/mol. (**B**) Three−dimensional structure of *RORC* (green) in complex with Celecoxib (blue). The protein is shown in green, the ligand in blue, binding residues in pink, and hydrogen bonds as red dashed lines. Interacting residues include Glu359 (2.8 Å), Gly470 (2.4 Å), Leu472 (2.8 Å), and Arg473 (1.9 Å). The second conformation of Celecoxib is shown. The binding affinity for this conformation is −8.8 kcal/mol.

Because *TCF7* lacks a canonical small-molecule binding pocket while *CLEC4D* could not find any potential drugs after interaction, docking was restricted to *RORC* (PDB: 7NPC), a nuclear receptor with a well-defined ligand-binding domain. Lovastatin showed strong binding (−9.0 kcal/mol) with robust hydrophobic interactions occupying the hydrophobic cleft (Figure 9A). Celecoxib’s top pose reached −9.7 kcal/mol, but the second conformation (−8.8 kcal/mol) was selected as it formed classical hydrogen bonds with key pocket residues (Figure 9B), providing additional stabilization. These low-affinity scores support direct *RORC* antagonism, offering pharmacological evidence for both drugs as potential COPD therapeutics.

### 2.11. qPCR Validation of Three Biomarkers

**Figure 10:**
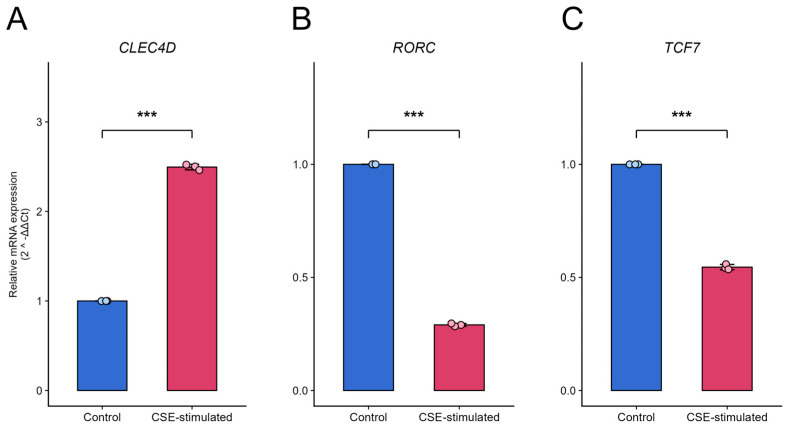
qRT−PCR validation of the three−gene signature in CSE−stimulated (COPD−like) cellular models. Expression levels of *CLEC4D*, *RORC*, and *TCF7* were measured by quantitative real−time PCR in THP−1−derived macrophages (for *CLEC4D*) and Jurkat T cells (for *RORC* and *TCF7*) following 24 h of stimulation with cigarette smoke extract: (**A**) *CLEC4D* expression was significantly upregulated in CSE−stimulated macrophages compared to control (*p* < 0.001). In contrast, (**B**,**C**) *RORC* and *TCF7* expression were significantly downregulated in CSE−stimulated Jurkat T cells compared to control (*p* < 0.001 for both). Data are presented as mean ± SD from three independent biological replicates. Statistical significance was determined using a two−tailed Student’s *t*-test. Significance: *** *p* < 0.001.

To validate the biological relevance of the identified three−gene signature, we established an in vitro COPD model using cigarette smoke extract (CSE)−stimulated human immune cell lines. Quantitative real−time PCR (qRT−PCR) analysis revealed distinct expression alterations consistent with our computational predictions (Figure 10A–C) with detailed data in Table S3. *CLEC4D* (Gene 1): In THP−1−derived macrophages, exposure to CSE resulted in a significant upregulation of *CLEC4D* mRNA levels compared to the control group (Relative Quantification [RQ] = 2.5 ± 0.07, *p* < 0.001), indicating a robust innate immune hyperactivation. *RORC* (Gene 2): Conversely, in Jurkat T cells, CSE treatment led to a drastic suppression of *RORC* expression (RQ = 0.29 ± 0.006, *p* < 0.001), suggesting an impairment of Th17−related transcriptional activity. *TCF7* (Gene 3): Similarly, *TCF7*, a critical regulator of T−cell stemness and longevity, was significantly downregulated in the CSE−treated group (RQ = 0.54 ± 0.01, *p* < 0.01).

### 2.12. Histological and Immunofluorescent Validation of TCF7

While our diagnostic model utilizes peripheral blood transcriptomics for noninvasive detection, COPD pathology is intrinsically driven by local airway inflammation. To determine if the systemic downregulation of *TCF7* mirrors the pulmonary immune microenvironment, validating its in situ protein expression is essential. We hypothesized that the depletion of *TCF7*-positive T cells in circulation reflects their recruitment to the lung and subsequent functional exhaustion. To bridge this gap, we performed hematoxylin and eosin (H&E) staining and immunofluorescence staining on human lung tissue to visualize *TCF7* loss specifically within infiltrating CD8^+^ T cells.

Prior to histological evaluation, we analyzed the baseline clinical characteristics of the tissue biopsy cohort (Table 3). As expected, the COPD group exhibited a significant decline in pulmonary function including FEV1/FVC and FEV1 percent predicted (*p* < 0.001) and a heavier smoking burden compared to controls. Crucially, blood routine examination revealed a significantly elevated neutrophil count in the patients with COPD (*p* < 0.001) with unchanged eosinophil levels, indicating a predominantly neutrophilic inflammatory phenotype.

**Figure 11:**
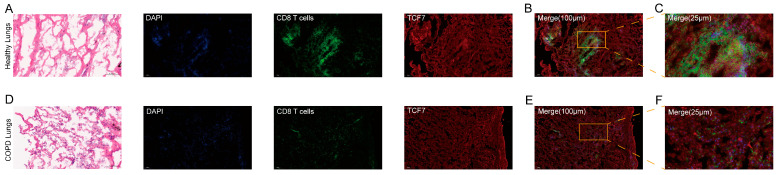
Histological and immunofluorescence validation of *TCF7* in human lung tissue: (**A**) H&E staining and separate immunofluorescence channels for DAPI (blue), CD8^+^ T cells (green), and *TCF7* (red) in healthy lung tissues. (**B**) Merged immunofluorescence image of healthy lung tissue. Scale bar is 100 μm. (**C**) Magnified merged immunofluorescence image of healthy lung tissue. Scale bar is 25 μm. (**D**) H&E staining and separate immunofluorescence channels for DAPI (blue), CD8^+^ T cells (green), and *TCF7* (red) in COPD lung tissues. (**E**) Merged immunofluorescence image of COPD lung tissue. Scale bar is 100 μm. (**F**) Magnified merged immunofluorescence image of COPD lung tissue. Scale bar is 25 μm.

H&E staining revealed evident structural differences between the healthy and COPD lung tissues, consistent with inflammatory remodeling (Figure 11A,D). Immunofluorescence co-staining demonstrated that CD8^+^ T cells (green) were more densely distributed and appeared relatively clustered in healthy lungs (Figure 11A–C). In contrast, the COPD samples exhibited reduced CD8^+^ cell density with a more sparse and dispersed distribution (Figure 11D–F). *TCF7* (red) signals were detectable in both groups; however, distinct spatial patterns were observed. In the healthy controls, *TCF7* displayed a relatively diffuse distribution and showed closer adjacency or apparent mixing with CD8 signals as observed in the merged and magnified images (Figure 11B,C). Conversely, the COPD tissues exhibited a more prominent punctate or granular pattern in the *TCF7* channel, with signals more frequently localized surrounding CD8^+^ cells and a reduced apparent overlap between the red and green channels (Figure 11E,F). These findings suggest altered CD8^+^ T-cell density and spatial organization of *TCF7*-associated signals in COPD lungs.

### 2.13. Pharmacological Validation of Celecoxib and Lovastatin In Vitro

**Figure 12:**
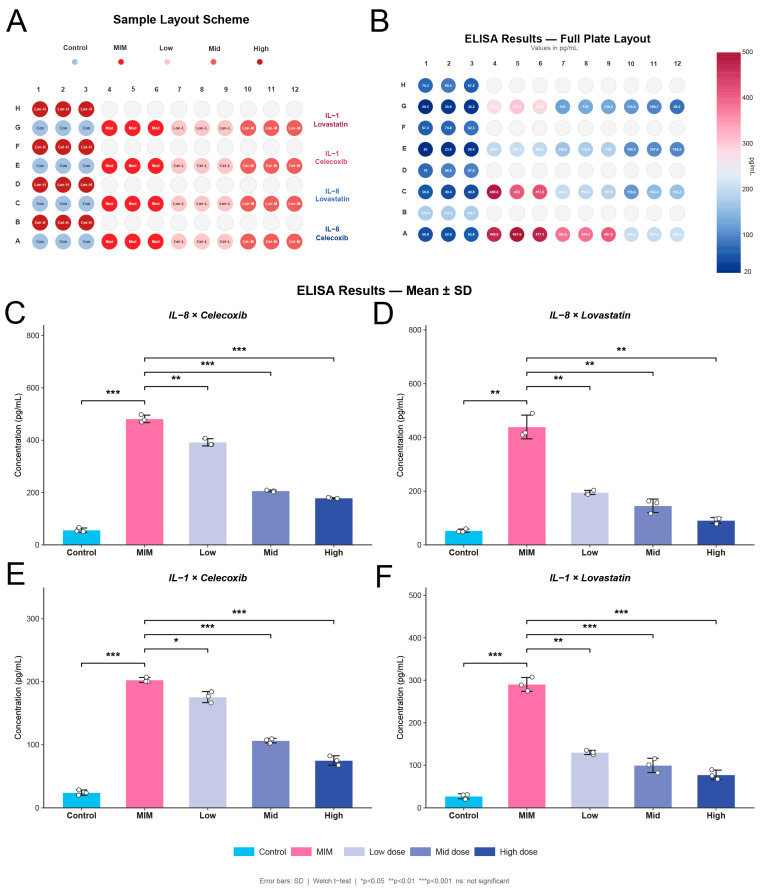
Validation of the anti-inflammatory efficacy of candidate drugs in LPS-primed and CSE-stimulated macrophages: (**A**) Schematic diagram of the 96-well plate layout showing the distribution of control, macrophage inflammatory model (MIM; LPS + CSE), and drug treatment groups across three independent replicates. (**B**) Heatmap representation of the raw concentration distribution across the 96-well plate, confirming consistent experimental conditions across all the wells. (**C**) Dose-dependent inhibition of IL-8 secretion by Celecoxib. Compared to the MIM group, Celecoxib treatment significantly reduced IL-8 levels in a concentration-dependent manner (*p* = 0.0015 for low dose, *p* < 0.001 for medium and high doses). (**D**) Dose-dependent inhibition of IL-8 secretion by Lovastatin. Lovastatin administration significantly attenuated IL-8 release across all the tested concentrations (*p* < 0.01 for all the doses). (**E**) Dose-dependent inhibition of IL-1β secretion by Celecoxib. Celecoxib robustly suppressed IL-1β levels, with medium and high doses achieving *p* < 0.001 compared to the MIM group. (**F**) Dose-dependent inhibition of IL-1β secretion by Lovastatin. Lovastatin significantly reduced IL-1β hypersecretion in a dose-dependent manner (*p* < 0.01 for low dose, *p* < 0.001 for medium and high doses). Significance: * *p* < 0.05, ** *p* < 0.01, *** *p* < 0.001.

To validate the therapeutic potential of the candidates predicted by our in silico molecular docking, we evaluated the anti-inflammatory effects of Celecoxib and Lovastatin using a “two-hit” (LPS priming followed by CSE challenge) macrophage inflammatory model. The experimental layout and corresponding raw absorbance distributions in the 96-well plate are illustrated in Figure 12A,B.

Consistent with COPD pathogenesis, the macrophage inflammatory model (MIM) group exhibited a dramatic hypersecretion of pro-inflammatory cytokines compared to the Control group, with IL-8 and IL-1β levels surging significantly (both *p* < 0.01, Welch *t*-test). Remarkably, drug interventions successfully reversed this innate immune hyperactivation in a strict dose-dependent manner. For Celecoxib treatment (Figure 12C,E), compared to the MIM group, the secretion of IL-8 was significantly attenuated across low to high concentrations (*p* = 0.0015 to *p* < 0.001), while IL-1β levels were robustly suppressed, particularly in the medium and high-dose groups (*p* < 0.001). Similarly, Lovastatin administration (Figure 12D,F) exerted potent immunomodulatory effects, significantly reducing the release of IL-8 (*p* < 0.01 across all the doses) and severely blunting IL-1β hypersecretion (*p* < 0.01 for low dose; *p* < 0.001 for medium and high doses). These robust in vitro functional data strongly corroborate our computational drug predictions, highlighting the feasibility of repurposing Celecoxib and Lovastatin to curb the pathogenic inflammatory cascade in COPD.

**Figure 13:**
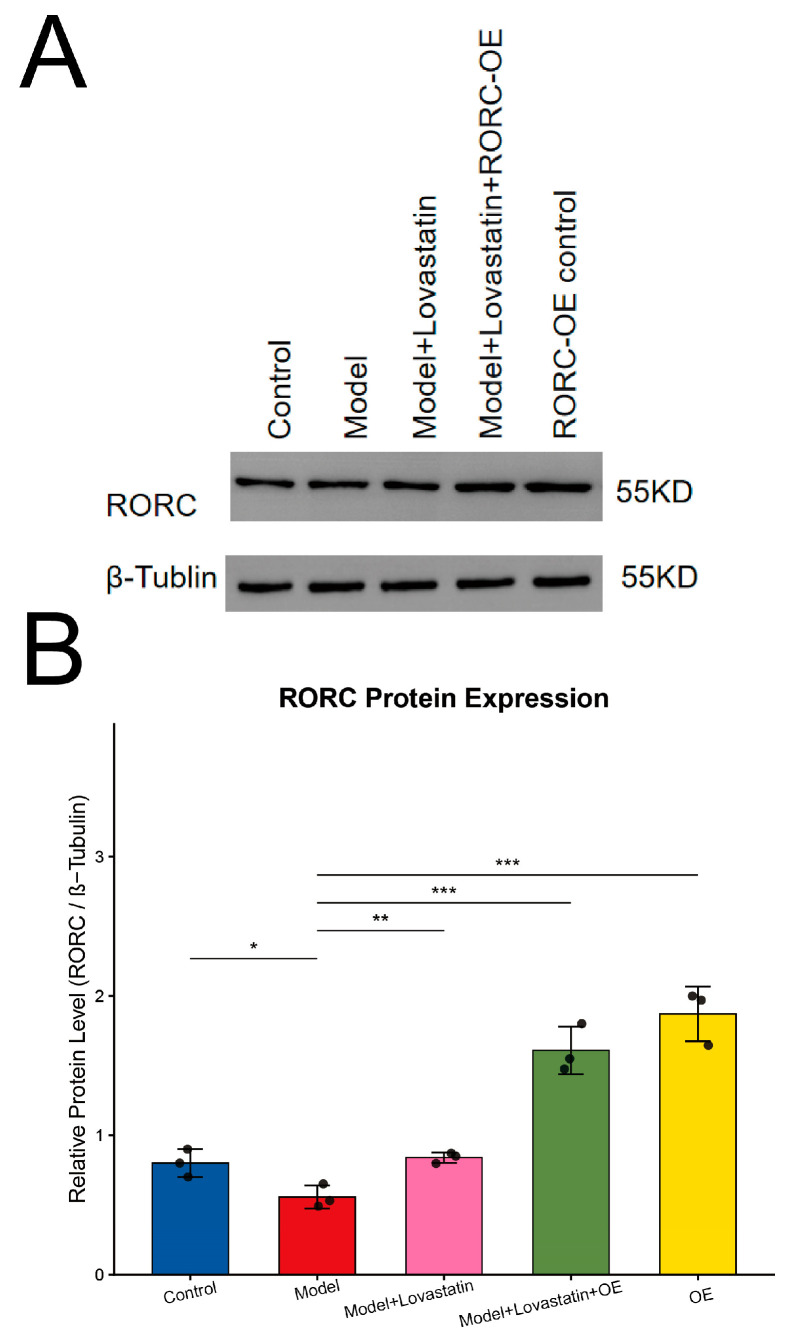
Pharmacological intervention restores *RORC* protein expression under inflammatory stress in macrophages: (**A**) Representative Western blot images of *RORC* and internal control β−tubulin in the THP−1−derived macrophages across five experimental conditions: Control, Model (LPS + CSE challenge), Model + Lovastatin, Model + Lovastatin + *RORC* overexpression (Model + Drug + OE), and OE alone. (**B**) Quantitative densitometric analysis of the *RORC* protein levels normalized to β−tubulin. The inflammatory model significantly suppressed *RORC* expression compared to the Control. Pharmacological treatment (Model + Lovastatin) successfully and significantly restored *RORC* protein levels. Data are presented as mean ± SD (*n* = 3 per group). Statistical significance was assessed using Student’s *t*−test. * *p* < 0.05, ** *p* < 0.01, *** *p* < 0.001.

To address whether the anti-inflammatory effects of the predicted drug are mediated through target modulation, we established an *RORC*-overexpression model in THP-1-derived macrophages (Figure 13A,B).

As illustrated by the quantitative Western blot analysis, the Model group exhibited a significant decrease in *RORC* expression compared to the Control group (*p* < 0.05). Crucially, pharmacological treatment via Lovastatin had successfully and significantly restored *RORC* protein levels (Model vs. Model + Lovastatin, *p* < 0.01), providing robust evidence of target modulation. Furthermore, forced overexpression of *RORC* (Model + Lovastatin + OE and OE alone) resulted in a highly significant surge in *RORC* protein levels compared to the Model baseline (both *p* < 0.001). These findings confirm highly efficient transfection and demonstrate that the therapeutic efficacy of Lovastatin involves the preservation of *RORC* expression under inflammatory stress.

### 2.14. TCF7 Depletion Downregulates Pro-Caspase-8 Expression in T Lymphocytes

**Figure 14:**
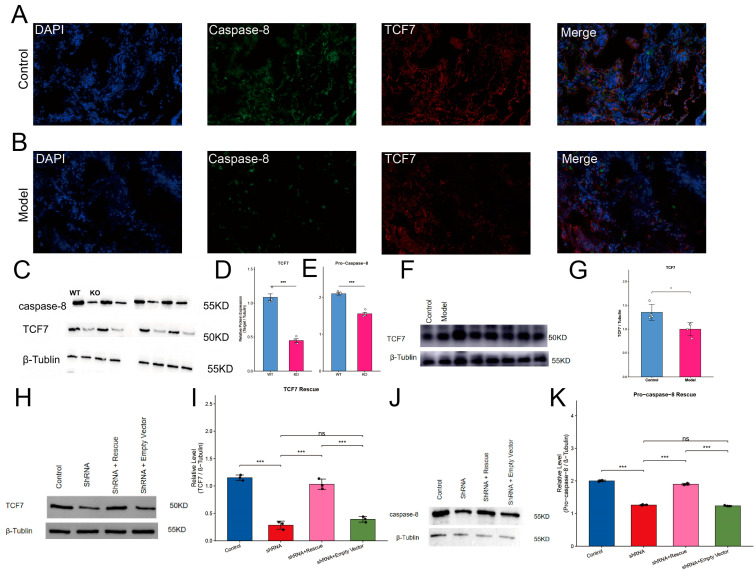
*TCF7* regulates pro−caspase−8 expression in T lymphocytes and is significantly reduced in COPD. (**A**) Immunofluorescence co−staining of control human lung tissue displaying separate channels for DAPI (blue), caspase−8 (green), *TCF7* (red), and the merged image. Scale bar is 50 μm. (**B**) Immunofluorescence co−staining of COPD human lung tissue displaying separate channels for DAPI (blue), caspase−8 (green), *TCF7* (red), and the merged image. Note the marked reduction in both *TCF7* and caspase−8 signals compared to the control. Scale bar is 50 μm. (**C**) Representative Western blot images of *TCF7* (50 kDa), pro−caspase−8 (55 kDa), and internal control β−tubulin (55 kDa) in wild type (WT) and *TCF7* knockout (KO) Jurkat T cells. (**D**) Quantitative densitometric analysis of *TCF7* protein levels comparing WT and KO groups. (**E**) Quantitative densitometric analysis of pro−caspase−8 protein levels comparing WT and KO groups. (**F**) Representative Western blot images of *TCF7* and β−tubulin in primary T lymphocytes isolated from the peripheral blood of healthy donors (Control) and patients with COPD (Model). (**G**) Quantitative densitometric analysis of *TCF7* protein levels in human primary T lymphocytes. (**H**) Representative Western blot images of *TCF7* and β−tubulin protein levels in Jurkat T cells across four experimental conditions including Control, shRNA, shRNA plus *TCF7* Rescue construct, and shRNA plus Empty Vector. (**I**) Quantitative densitometric analysis of *TCF7* protein levels across the four experimental rescue groups. (**J**) Representative Western blot images of pro−caspase−8 and β−tubulin protein levels across the same four experimental conditions in Jurkat T cells. (**K**) Quantitative densitometric analysis of pro−caspase−8 protein levels across the four experimental rescue groups. Data in the bar charts are presented as mean ± SD (*n* = 4 for primary human cells, *n* = 3 for cell line experiments). Statistical significance was assessed using Student’s *t* test with Welch’s correction where appropriate (* *p* < 0.05, *** *p* < 0.001, ns indicates not significant).

To validate the clinical coexistence of *TCF7* and caspase-8 dysregulation in the target organ, immunofluorescence co-staining of human lung tissue sections (20× magnification) was performed. Control tissues revealed prominent *TCF7*-positive signals (red) and caspase-8 signals (green) with clear overlap in the merged images (Figure 14A). In contrast, *TCF7* and caspase-8 immunoreactivity became markedly sparse and diffuse in COPD lung sections (Figure 14B). This spatial loss of *TCF7* co-occurred with an altered caspase-8 distribution within the pulmonary microenvironment, providing direct histological evidence for the concurrent downregulation of this axis in vivo.

Given that *TCF7* is downregulated in COPD and predominantly localized in the lymphoid lineage based on our single-cell analysis, we sought to elucidate its downstream molecular consequences. We established a *TCF7*-deficient model in the human Jurkat T-cell line and assessed protein expression via Western blotting (Figure 14C). Quantitative densitometric analysis confirmed the highly efficient suppression of *TCF7*, with its protein expression drastically reduced in the knockout (KO) group compared to the wild type (WT) controls (*p* < 0.001) (Figure 14D). To investigate its impact on cell fate decisions, we evaluated the expression of pro-caspase-8 (55 kDa), a critical initiator zymogen in the extrinsic apoptotic pathway. The targeted depletion of *TCF7* led to a parallel and highly significant downregulation of pro-caspase-8 protein levels (*p* < 0.001) (Figure 14E).

To validate this molecular alteration in a clinically relevant context, we isolated primary T lymphocytes from the peripheral blood of healthy donors and patients with COPD. Consistent with our findings in cell lines, the Western blot analysis demonstrated a significant downregulation of *TCF7* protein levels in COPD-derived primary T cells compared to healthy controls (*p* < 0.05) (Figure 14F,G). This directly establishes the translational relevance of this molecular axis in human disease.

To establish the causal relationship between *TCF7* and pro-caspase-8, we performed a genetic rescue experiment. Jurkat T cells were transduced with control shRNA, *TCF7*-targeting shRNA, or co-transduced with *TCF7*-shRNA alongside a full-length human *TCF7* construct (Rescue group). Targeted *TCF7* knockdown led to a drastic abrogation of *TCF7* protein levels. Remarkably, the re-introduction of *TCF7* in the Rescue group successfully restored *TCF7* expression (*p* < 0.001 compared to shRNA alone and the shRNA plus Empty Vector baseline) (Figure 14H,I). The addition of the empty vector did not significantly alter the *TCF7* knockdown baseline (ns). Regarding the downstream effector, Western blot analysis of pro-caspase-8 revealed a consistent biological trend (Figure 14J). While *TCF7* knockdown noticeably reduced pro-caspase-8 band intensity, the Rescue group exhibited a clear and statistically significant restoration of pro-caspase-8 expression compared to the stringent dual-transfected negative control (*p* < 0.001) (Figure 14K). This rigorous negative control strictly rules out nonspecific plasmid effects and provides compelling functional evidence that *TCF7* causally governs pro-caspase-8 expression. The pathological loss of *TCF7* in COPD likely disrupts this critical caspase-8-dependent survival network in T lymphocytes.

## 3. Discussion

The present study outlines a potential immune-dysregulation framework for COPD by integrating large-scale peripheral blood transcriptomics with machine learning feature selection, immune deconvolution, regulatory inference, and single-cell resolution mapping, followed by preliminary experimental validation. COPD is increasingly viewed as a heterogeneous, immune-mediated inflammatory condition in which systemic immune perturbations reflect and potentially inform lung pathology and clinical stratification [19,20,21,22]. In this context, blood-based transcriptional biomarkers are attractive because they are scalable, minimally invasive, and can capture robust molecular associations linked to airway and parenchymal disease [23]. By leveraging a large training cohort and multiple independent validation datasets, including lung tissue, we identified a three-gene signature (*CLEC4D*, *RORC*, *TCF7*) that showed consistent directionality across cohorts and provided clinically oriented performance in ROC analyses and a nomogram with supportive calibration and decision-curve characteristics. These findings align with prior COPDGene-based work demonstrating that peripheral blood transcriptomic programs can stratify airway disease phenotypes and COPD risk, supporting the translational potential of blood-derived molecular classifiers in COPD [24,25].

At the systems level, the differentially expressed gene landscape and enrichment profile converged on innate immune activation, pathogen-sensing receptor functions, and granule-associated cellular components. This pattern is biologically congruent with contemporary COPD immunopathogenesis models that emphasize persistent myeloid activation, neutrophil-associated tissue injury, and dysregulated innate host-defense programs [24,25]. Within the three-gene signature, *CLEC4D* emerged as the component most tightly aligned with this innate axis. The induction of *CLEC4D* following cigarette smoke extract stimulation further supports the interpretation that it likely reflects a smoke-responsive innate activation state. To move beyond descriptive in silico predictions and establish functional relevance, we performed in vitro pharmacological validation using a clinically relevant two-hit (LPS/CSE) macrophage model. Our ELISA results demonstrated that Celecoxib and Lovastatin, the top drug candidates identified through molecular docking, dose-dependently attenuated the hypersecretion of IL-8 and IL-1β. Although immediate clinical applicability will require rigorous prospective validation, these in vitro data provide a critical proof-of-concept that the identified transcriptomic signature corresponds to a pharmacologically targetable axis that may curb innate immune hyperactivation in COPD. To further investigate whether the anti-inflammatory effects of drugs are mediated through *RORC*, we performed *RORC* overexpression experiments in THP-1-derived macrophages. *RORC* overexpression alone markedly increased *RORC* protein levels, and when combined with drug treatment, it further enhanced *RORC* expression compared to drug treatment alone. These findings support a model in which Celecoxib and Lovastatin act, at least in part, through *RORC* to attenuate innate immune hyperactivation in COPD. Notably, single-cell studies of COPD lung tissue consistently highlight remodeling of myeloid populations, providing cellular context for why a myeloid-enriched marker such as *CLEC4D* is detectable in both the circulation and the diseased lung environment [26,27,28,29].

Beyond innate activation, our immune functional profiling and cell–cell communication analyses suggest a broader, disease-wide immunoregulatory shift in COPD characterized by impaired antigen presentation and T-cell priming capacity, alongside enhanced inhibitory signaling. Specifically, HLA-related signatures and T-cell co-stimulation were suppressed while APC and T-cell co-inhibition were increased, consistent with an immune ecosystem that is inflamed yet functionally constrained. This pattern resonates with emerging conceptual frameworks that reposition COPD among immune-mediated inflammatory diseases, emphasizing persistent immune pathway engagement at sites of tissue damage coupled with maladaptive regulatory circuits [30,31,32,33]. The observed reduction in T follicular helper signatures in blood is also notable in light of evidence that human lung dendritic cells can orchestrate Tfh polarization and tertiary lymphoid structure formation in COPD lungs, suggesting that compartmental redistribution and local immune architecture remodeling may coexist with systemic signatures of impaired adaptive coordination [34]. In our CellChat analysis, the global reduction in interaction complexity together with loss of homeostatic pathways and consolidation of inflammatory signaling is consistent with a transition from flexible immune homeostasis toward a more rigid, pathologically stabilized network state; the prominence of MIF-centered signaling is biologically consistent given the pleiotropic role of MIF in chronic lung disease biology and immune cell recruitment and activation programs [35]. Collectively, these multi-layer observations support a whole-disease view in which COPD is not only an innate inflammatory disorder but also a disorder of impaired immune coordination and regulated immune suppression.

The adaptive immune dimension of this signature is anchored by *RORC* and *TCF7*, which provide mechanistic interpretability to the observed immune functional landscape. *RORC*, a lineage-defining transcriptional regulator for Th17-associated programs, was consistently downregulated across datasets and suppressed by cigarette smoke extract stimulation in Jurkat T cells [36,37,38]. *TCF7*, which encodes TCF-1, adds a complementary layer by indexing T-cell durability and differentiation potential under chronic inflammatory stress [39,40]. To directly address the mechanistic consequences of *TCF7* downregulation and establish causality, we performed genetic rescue experiments in Jurkat T cells. *TCF7* knockdown significantly reduced both *TCF7* and pro-caspase-8 protein levels. Re-expression of *TCF7* in the rescue group successfully restored *TCF7* expression and significantly increased pro-caspase-8 levels compared to the stringent dual-transfected empty vector control. These results provide direct functional evidence that *TCF7* positively regulates pro-caspase-8 expression. Crucially, to bridge the translational gap between reductionist cell lines and human disease, we validated this axis in patient-derived primary T lymphocytes, confirming that *TCF7* is significantly depleted in the circulating functional immune cells of patients with COPD. Recent landmark studies have established TCF-1 as a central transcriptional network driving the effector versus exhausted T-cell fate decision [39]. Concurrently, caspase-8 has been identified not merely as an apoptotic initiator but as a fundamental molecular switch regulating immune cell survival and inflammatory cell death [41]. Together, these findings support a model in which the systemic loss of *TCF7* disrupts caspase-8-dependent survival signaling, potentially accelerating T-cell exhaustion in COPD. Importantly, the lung tissue cohort and immunofluorescence findings extend this adaptive narrative beyond blood. Immunofluorescence co-staining of human lung tissue further revealed that *TCF7* and caspase-8 were co-localized in control lungs, whereas both signals were markedly reduced and spatially disrupted in COPD tissues. This in situ observation perfectly corroborates our ex vivo primary cell findings, suggesting that the *TCF7*-caspase-8 axis is severely disrupted at the protein level within the pulmonary microenvironment.

Taken together, the three-gene signature reflects complementary but biologically connected aspects of COPD pathogenesis rather than three isolated biomarkers. *CLEC4D* primarily represents innate immune and myeloid activation, consistent with the neutrophil- and macrophage-dominant inflammatory milieu in COPD, whereas *RORC* and *TCF7* indicate adaptive immune dysregulation, including impaired lymphocyte differentiation, persistence, and functional homeostasis under chronic inflammatory stress. In this context, the disrupted *TCF7*-caspase-8 axis may contribute to defective T-cell maintenance and sustained immune imbalance in COPD. From a translational perspective, this panel is not intended to replace spirometry, but may serve as a complementary blood-based molecular tool for supportive diagnosis, risk stratification, and immune endotype assessment.

Several limitations should be considered when interpreting these results. First, while the primary differential expression analysis did not explicitly adjust for covariates, a sensitivity analysis incorporating inferred sex and age confirmed that the three-gene signature remained robust. Second, the bulk blood transcriptomic signals necessarily reflect both cell-intrinsic regulation and shifts in leukocyte composition. Third, public datasets incompletely annotate several clinically relevant covariates that may influence gene expression patterns, including smoking exposure, exacerbation status, inhaled therapies, medication use, and coexisting comorbidities; therefore, residual confounding cannot be fully excluded, and prospective cohorts with harmonized metadata will be important [42,43,44]. Fourth, although our newly added functional assays advanced from immortalized cell lines to patient-derived primary cells and genetic rescue models, direct in vivo validation of the proposed mechanisms is still lacking. Therefore, the causal roles of the *TCF7*-caspase-8 axis and related immune pathways should be further evaluated in targeted animal models in future studies. Additionally, while our *RORC* overexpression data support that Celecoxib and Lovastatin act through *RORC* preservation, direct biophysical evidence of drug-protein binding (e.g., cellular thermal shift assay or surface plasmon resonance) is still lacking and should be addressed in future investigations. Fifth, the number of lung tissue samples for immunofluorescence was limited, and the potential influence of distinct inflammatory phenotypes or inhaled therapies on *TCF7* expression could not be fully stratified. Although our clinical biopsy cohort exhibited a neutrophilic profile consistent with our transcriptomic findings, these histological results should be interpreted cautiously. Future studies using larger cohorts and spatial transcriptomics are needed to dissect this local heterogeneity. Sixth, while the three-gene model is intentionally parsimonious, integrating it with established clinical variables and imaging-based phenotypes may further improve robustness across COPD endotypes [45,46].

## 4. Materials and Methods

### 4.1. Data Sources

Gene expression datasets were retrieved from the Gene Expression Omnibus (GEO) repository (Table 4). Datasets were included if they met all of the following criteria: (i) human samples with clearly defined COPD and non-COPD control groups were available; (ii) transcriptomic data and corresponding platform annotations were publicly accessible; (iii) the sample source and disease status could be clearly determined from the original GEO metadata or accompanying publications; and (iv) the dataset provided either sufficient sample size for biomarker discovery or specific value for independent validation, regulatory analysis, or single-cell localization. Datasets or samples were excluded if they lacked clear case or control annotations, contained duplicated samples, involved major concomitant diseases that could not be separated from COPD status, or included treatment-related confounders that could substantially affect gene expression.

Among the eligible datasets, GSE71220 was selected as the training cohort because of its relatively large sample size and detailed clinical annotations, enabling robust differential expression and machine learning-based feature selection. To reduce medication-related transcriptional confounding, samples annotated as “statin_pt” were excluded a priori from GSE71220. After filtering, 405 COPD and 44 control blood samples were retained for analysis. GSE42057 (GPL570; 94 COPD and 42 controls) was used as an internal validation cohort to assess reproducibility within peripheral blood. GSE24709 (GPL9040) was used for miRNA-related regulatory analysis; samples with lung cancer were excluded before analysis, leaving 24 COPD and 19 control samples. GSE249584 (GPL24676; 8 COPD and 7 controls) was included for single-cell RNA-seq analysis to determine the cellular localization of the identified biomarkers.

To further evaluate cross-platform and cross-tissue robustness, three independent external validation cohorts were included: GSE56766 (GPL570; 49 COPD and 29 controls), GSE306950 (GPL30209; 25 COPD and 10 controls), and GSE106986 (GPL13497; 14 COPD and 5 controls). The first two datasets were derived from peripheral blood, whereas GSE106986 used lung tissue samples, thereby providing complementary tissue-level validation. All the datasets were processed independently using platform-appropriate normalization methods, and no cross-dataset batch correction was performed in order to preserve platform-specific biological signals and avoid introducing technical distortion. Patient sex was determined for three GEO datasets (GSE249584, GSE56766, and GSE306950) by extracting expression values of sex-specific marker genes (*XIST* and *RPS4Y1*), classifying samples using median-based thresholds with manual curation applied where needed. For the scRNA-seq dataset (GSE249584), pseudobulk aggregation was performed per donor prior to classification; raw FPKM values were used directly for GSE306950 to preserve interpretability without log-transformation. Detailed results for the gender are provided in Figures S2–S4.

For preprocessing, Affymetrix raw data were normalized using the Robust Multi-array Average (RMA) algorithm. RNA-seq data utilized log2(FPKM + 1) transformation, and other datasets utilized log2-transformed Series Matrices. To preserve platform-specific signals and avoid distortion, the datasets were analyzed independently without cross-dataset batch correction.

### 4.2. Differential Expression Analysis

Differentially expressed genes (DEGs) between the patients with COPD and healthy controls in the training cohort GSE71220 were identified using the limma R package (v3.54.0) [52]. Genes with |log_2_FC| > 0.3 and adjusted *p*-value (Padj) < 0.05 (Benjamini–Hochberg correction) were considered statistically significant [53]. Visualization was performed using ggplot2 (v3.4.0) and ComplexHeatmap (v2.14.0). Unless otherwise specified, statistical comparisons between groups were conducted using the two-tailed Wilcoxon rank-sum test.

### 4.3. Functional Enrichment and PPI Network

The GO and KEGG enrichment analyses were performed with clusterProfiler (v4.6.0) [54], with significance set at *p*-value < 0.05. To visualize the functional relationships among enriched terms, a network of enriched GO and KEGG terms was constructed using Metascape (https://metascape.org (accessed on 14 Janurary 2026)). Significantly enriched terms (*p* < 0.05) were clustered based on gene overlap similarity. For protein–protein interaction (PPI) network analysis, the STRING database (https://string-db.org (accessed on 14 Janurary 2026)) was used to retrieve known and predicted protein interactions for the DEGs, with a medium confidence score ≥ 0.4 [55]. The resulting network was imported into Cytoscape (v3.9.1) for visualization and identification of hub genes based on degree centrality.

### 4.4. Feature Selection by Machine Learning

The Boruta algorithm (R package Boruta v7.0.0) [56] was applied with parameters maxRuns = 300 and *p*-value = 0.01 for all-relevant feature selection. Feature importance was visualized using ImageGP (v1.0). The Least Absolute Shrinkage and Selection Operator (LASSO) regression was performed with glmnet (v4.1-7) using 5-fold cross-validation; non-zero coefficient genes at optimal λ were retained [57]. Robust biomarkers were defined by intersecting Boruta- and LASSO-selected genes using ggvenn (v0.1.9).

### 4.5. Biomarker Evaluation and Predictive Model Construction

Biomarker expression differences were evaluated in both training and validation sets via the Wilcoxon test. Diagnostic accuracy was assessed using ROC curve analysis with pROC (v1.18.4) retaining biomarkers with AUC > 0.60. A nomogram was constructed with rms (v6.7-1), and calibration was evaluated using ResourceSelection (v1.2-8). A decision curve analysis (DCA) assessed clinical utility [58,59].

### 4.6. External Validation Analysis

In this study, to rigorously evaluate the external validity and cross-platform robustness of our candidate biomarkers, we analyzed three independent public datasets. These datasets encompassed whole blood samples from patients with COPD and healthy individuals (GSE56766 and GSE306950), as well as lung tissue from patients with critical COPD (GSE106986). An independent analysis strategy was employed for each dataset to preserve platform-specific signal characteristics and avoid technical biases from cross-platform normalization. For each validation dataset, the expression of three candidate biomarkers *RORC*, *CLEC4D*, and *TCF7* was compared between COPD and control groups using the non-parametric Wilcoxon rank-sum test. The diagnostic performance was assessed by Receiver Operating Characteristic (ROC) curve analysis, implemented with the pROC package in R. To quantify diagnostic accuracy, the Area Under the Curve (AUC) and its 95% confidence interval (CI) were calculated. The magnitude of expression differences was evaluated by calculating Cohen’s d as a measure of effect size. A *p*-value < 0.05 was considered statistically significant. For visualization, expression distributions were depicted using violin plots with embedded box plots, and ROC curves were smoothed via Locally Estimated Scatterplot Smoothing (LOESS, span = 0.3) to improve interpretability [60].

### 4.7. Regulatory Network Analyses

TF–miRNA co-regulatory networks were constructed via NetworkAnalyst 3.0 (ENCODE database) [61] and visualized with Cytoscape using cohort GSE24709. The predicted miRNA–gene interactions were retrieved from TargetScan [62], intersected with DEmiRNAs using ggvenn, and visualized in Cytoscape. Gene–gene interaction networks were predicted using GeneMANIA [63].

### 4.8. Immune Infiltration and Functional Analysis

Immune cell infiltration was quantified using ssGSEA implemented in GSVA (v1.48.0) [64]. Heatmaps were generated with pheatmap (v1.0.12). Pairwise correlations among 28 immune cell types, and between biomarkers and immune infiltration, were analyzed with psych (v2.3.9) and visualized in ggplot2. Thirteen immune function gene sets were used to assess immune activity. HLA gene–biomarker correlations were calculated with the cor function in R.Gene Set Enrichment Analysis (GSEA) was performed to further enrich the function of 3 biomarkers with clusterProfiler against the MSigDB c2.cp.kegg_legacy.v2024.1.Hs.symbols.gmt gene set [65].

### 4.9. Single-Cell RNA-Sseq Analysis

QC was performed in Seurat (v4.3.0) [59], retaining cells expressing ≥200 genes, ≤4000 genes per cell, ≤60,000 counts, and <25% mitochondrial content. Highly variable genes (top 2000) were identified via FindVariableFeatures. PCA was conducted with RunPCA, and significant PCs were determined by Jackstraw tests (*p* < 0.05). Clustering was performed using FindNeighbors/FindClusters (resolution = 1.3) and visualized with UMAP [66]. Marker genes were identified with FindAllMarkers (logFC ≥ 0.5, min.pct ≥ 0.2). Key cell types were defined by differential abundance (Wilcoxon test) and differential expression of prognostic genes. DEGs within clusters were identified with FindMarkers and visualized in ggplot2. Cell–cell communication was inferred using CellChat (v1.5.0) [67] and validated with celltalker.

### 4.10. Drug Prediction and Molecular Docking

To identify candidate drugs capable of reversing the COPD signature, a dual-screening strategy was employed. First, differentially expressed genes were queried against the Connectivity Map (cMap) database (https://clue.io/ (accessed on 17 Janurary 2026)) [66]). [68] To prioritize compounds with therapeutic potential and defined mechanisms, the cMap results were filtered to retain only those with a negative raw connectivity score (cs < 0, indicating a trend of disease signature reversal) while strictly excluding unannotated compounds (MOA = −666) [69]. Concurrently, the core biomarkers were submitted to the Enrichr platform to identify drug–gene interactions via the Drug Signatures Database (DSigDB v1.0). Statistical significance in DSigDB was strictly defined by a False Discovery Rate (FDR) < 0.05. Overlapping candidate compounds from both databases were then prioritized for downstream structural validation. Given that *TCF7* lacks a canonical small-molecule binding pocket and *CLEC4D* yielded no overlapping targeted drugs, molecular docking was performed solely for *RORC* (PDB ID: 7NPC), which harbors a well-defined ligand-binding domain. The protein structure was prepared in PyMOL (v2.4.0) by removing water molecules and native organic ligands. The 3D structures of the candidate compounds (Lovastatin and Celecoxib) were retrieved from the PubChem database, energy-minimized in Chem3D (v19.0), and prepared in AutoDock Tools (v1.5.7) with the addition of polar hydrogens and computation of Gasteiger charges. Docking was executed using AutoDock Vina (v1.1.2) with a comprehensive grid box covering the binding cavity (center X = 2.939, Y = −39.831, Z = −4.505). The optimal binding conformation was determined by evaluating both the lowest binding affinity (kcal/mol) and the presence of stable hydrogen-bonding networks within the active site.

### 4.11. Validation of Biomarkers via Quantitative Real-Time PCR

To provide preliminary experimental validation of the identified biomarkers under a COPD-relevant insult, we performed cigarette smoke extract (CSE) stimulation followed by quantitative real-time PCR (qRT-PCR) in human immune cell lines [70]. THP-1 monocytes and Jurkat T cells were cultured in RPMI-1640 (Gibco, Grand Island, NY, USA; Cat. No. 11875093) supplemented with 10% fetal bovine serum (Gibco; Cat. No. 10099141) and 1% penicillin/streptomycin (Gibco; Cat. No. 15140122) at 37 °C with 5% CO2; THP-1 cells were differentiated into macrophage-like cells using PMA (100 nM, 24 h) and then rested in PMA-free medium for 24 h before stimulation [71]. CSE was freshly prepared by bubbling smoke from one commercial cigarette through 10 mL serum-free RPMI-1640 (Gibco, Grand Island, NY, USA; Cat. No. 11875093), filtered through a 0.22 µm membrane as 100% CSE stock, and diluted to a final concentration of 2% (v/v) for cell treatment; cells were exposed to CSE for 24 h, while control cells received an equal volume of vehicle. Total RNA was extracted using TRIzol reagent (Invitrogen, Carlsbad, CA, USA; Cat. No. 15596026), and 1 µg RNA was reverse-transcribed into cDNA using a reverse transcription kit with genomic DNA removal (Takara Bio, Shiga, Japan; Cat. No. RR047A) [72].qRT-PCR was performed in technical triplicate using SYBR Green chemistry (Bio-Rad, Hercules, CA, USA; Cat. No. 1725124) on a QuantStudio real-time PCR system (Thermo Fisher Scientific, Waltham, MA, USA) with the following cycling program: 95 °C for 30 s, then 40 cycles of 95 °C for 5 s and 60 °C for 34 s, followed by melt-curve analysis to confirm specificity. Relative expression levels were calculated using the 2^−ΔΔCt^ method, normalized to GAPDH, based on at least three independent biological replicates (*n* = 3), and statistical significance between groups was assessed using a two-tailed Student’s *t*-test with *p* < 0.05 considered significant [73].

### 4.12. Cell Culture, Sequential Inflammatory Stimulation, and ELISA

To establish a clinically relevant in vitro model mimicking the complex inflammatory microenvironment of COPD, a sequential stimulation protocol was employed using the THP-1 human monocytic cell line. Initially, all the cells were differentiated into macrophage-like cells by incubation with 100 nM Phorbol 12-myristate 13-acetate (PMA; Cat. No. 524400-1MG, Merck, Darmstadt, Germany). Following differentiation, the culture supernatants were discarded. For the inflammatory priming phase, cells in the Model (M) and Drug intervention groups were incubated with 100 ng/mL Lipopolysaccharide (LPS; Cat. No. L4391-1MG, Merck) for 24 h to simulate bacterial colonization, whereas the Control group received standard culture medium without LPS.

The culture supernatants were removed again to initiate the challenge phase. To mimic smoking-induced acute inflammatory bursts, cells in the Model group were exposed to cigarette smoke extract (CSE). Concurrently, cells in the Drug intervention groups were co-treated with CSE and varying concentrations of the candidate compounds: Celecoxib (1, 25, and 50 μM; Cat. No. T92566, MedMol/Shanghai Yuanye Bio-Technology, Shanghai, China) or Lovastatin (1, 10, and 25 μM; Cat. No. S25584, MedMol). The Control group cells were maintained in fresh medium throughout this phase [74,75].

After 24 h of CSE and drug co-incubation, the cell culture supernatants from all the groups were collected and centrifuged to remove cellular debris. The secretion levels of pro-inflammatory cytokines were quantified using specific human ELISA kits strictly according to the manufacturer’s instructions. Interleukin-8 (IL-8) was measured using the EasyGo Human IL-8 One-Step ELISA Kit (Cat. No. EK108EG, Multi Sciences, Hangzhou, China), and Interleukin-1β (IL-1β) was evaluated utilizing the Human IL-1β ELISA Kit (Cat. No. EK101B, Multi Sciences). Optical density was measured at 450 nm using a microplate reader, and cytokine concentrations (pg/mL) were calculated based on the standard curves.

### 4.13. Histological and Immunofluorescence Analysis

Human lung tissue specimens were obtained from patients undergoing bronchoscopy for benign pulmonary nodules at the Department of Respiratory Medicine, The First Affiliated Hospital of Kunming Medical University. A total of 30 patients with benign nodules were initially enrolled, consisting of 15 patients with comorbid COPD and 15 control subjects without COPD. Detailed baseline clinical characteristics of the enrolled subjects were retrospectively collected, encompassing demographic data (age, sex, BMI), smoking history (pack-years and status), comprehensive pulmonary function tests (FEV1/FVC and FEV1%predicted), GOLD staging, and peripheral blood inflammatory cell counts like neutrophils and eosinophils. The patients with an acute exacerbation of COPD (AECOPD) within three months prior to sampling or those with concomitant asthma were strictly excluded to avoid confounding acute inflammatory signals. From this cohort, normal-appearing lung tissues adjacent to the benign nodules were collected from 6 randomly selected individuals (3 patients with COPD and 3 non-COPD controls) for downstream validation. The freshly collected specimens were immediately fixed in 4% paraformaldehyde and embedded in paraffin. For histological assessment, sections were deparaffinized, rehydrated, and stained with hematoxylin and eosin (H&E) according to standard protocols to visualize pathological changes. To investigate the colocalization of *TCF7* expression within CD8^+^ T cells, immunofluorescence staining was performed. Tissue sections were deparaffinized, rehydrated, and subjected to heat-induced antigen retrieval with citrate buffer. The sections were then blocked with 5% donkey serum (Shanghai Fine Bio Tech Inc., Shanghai, China; Cat. No. FK4622) and incubated overnight at 4 °C with primary antibodies against *TCF7* (Rabbit anti-human; Shanghai Fine Bio Tech Inc., Shanghai, China; Cat. No. FA02441) and CD8 (Mouse anti-human; Shanghai Fine Bio Tech Inc., Shanghai, China; FA00007). Following three washes with PBS, the sections were incubated with the corresponding species-specific fluorophore-conjugated secondary antibodies (Alexa Fluor 488-conjugated anti-mouse and Alexa Fluor 594-conjugated anti-rabbit; both from Invitrogen, Carlsbad, CA, USA; Cat. Nos. A11001 and A11012). Nuclei were counterstained with DAPI. Fluorescence signals were captured using a ZEISS LSM980 confocal microscope. Specifically, CD8^+^ T cells were identified by green fluorescence at an emission wavelength of 520 nm, while *TCF7* expression was visualized as red fluorescence at 570 nm [76]. For *TCF7* and caspase-8 co-localization, sections were incubated with rabbit anti-*TCF7* (same as above) andmouse anti-caspase-8 (Santa Cruz Biotechnology, Dallas, TX, USA; Cat. No. sc-56056). After washing, sections were incubated with Alexa Fluor 488-conjugated anti-mouse (green; Invitrogen, Carlsbad, CA, USA; Cat. No. A11001) and Alexa Fluor 594-conjugated anti-rabbit (red; Invitrogen; Cat. No. A11012) secondary antibodies.

### 4.14. RORC Overexpression in THP-1-Derived Macrophage

To investigate whether the anti-inflammatory effects of the candidate drugs are mediated through *RORC*, genetic overexpression was performed in THP-1-derived macrophages. Lovastatin was chosen for this mechanistic study and used at a concentration of 10 μM. The human *RORC* ORF clone (ROR gamma (*RORC*) Human Tagged ORF Clone, transcript variant 2, NM_001001523, encoding the RORγt isoform) was obtained from OriGene (Cat. No. RC224338). THP-1 cells were first differentiated into macrophages with 100 nM PMA for 24 h, then transfected with 2 μg of the *RORC* overexpression plasmid or empty vector control using Lipofectamine 3000 (Invitrogen, Cat. No. L3000015) according to the manufacturer’s protocol. After 48 h, the cells were subjected to sequential LPS/CSE stimulation and drug treatment as described in Section 4.12. *RORC* overexpression efficiency and drug response were confirmed by Western blotting.

### 4.15. Isolation of Primary Human T Lymphocytes

To biologically validate the specific transcriptomic findings in a patient-derived context, peripheral blood samples were collected from healthy donors and patients with COPD. Peripheral blood mononuclear cells (PBMCs) were initially isolated via density gradient centrifugation using Ficoll–Paque. Subsequently, primary human T lymphocytes were purified from the PBMCs by adhering macrophages to culture flasks for 2 h and collecting the non-adherent T lymphocyte suspension according to standard protocols. The isolated primary T cells were then immediately lysed for Western blot analysis.

### 4.16. Western Blotting Analysis

To elucidate the downstream molecular mechanisms, Western blot analysis was conducted utilizing THP-1-derived macrophages, Jurkat T cells (including WT, KO, and Rescue models), and primary human T lymphocytes. Total proteins were extracted using RIPA lysis buffer supplemented with protease inhibitors. Protein concentrations were determined using a BCA protein assay kit. Equal amounts of protein lysates were resolved on 4–20% precast polyacrylamide gels (Cat. No. P06, willget/Shanghai Wenyuange Biological Technology, Shanghai, China) and subsequently transferred onto PVDF membranes (Millipore, Burlington, MA, USA; Cat. No. IPVH00010) using a 50X universal electrophoresis and electrotransfer buffer system (Cat. No. P08, willget). The membranes were blocked with 5% non-fat milk at room temperature for 1 h and then incubated overnight at 4 °C with the following primary antibodies: anti-*TCF7* (1:2000; Cat. No. 14464-1-AP, Proteintech), anti-Caspase 8/P43/P18 (1:2000; Cat. No. 13423-1-AP, Proteintech), anti-*RORC* (1:1000; Cat. No.29910-1-AP, Proteintech), and anti-Beta Tubulin (1:2000; Cat. No. 10094-1-AP, Proteintech) as the internal loading control. Following three washes with TBST, the membranes were incubated with HRP-conjugated secondary antibodies (1:15,000 dilution) for 1 h at room temperature. Protein bands were visualized using an enhanced chemiluminescence (ECL) detection system (Thermo Fisher Scientific, Waltham, MA, USA; Cat. No. 34580). The quantitative analysis of protein band gray values was performed using the ImageJ software (version 1.54f).

### 4.17. TCF7 Knockdown and Rescue Experiments in Jurkat T Cells

To establish the causal relationship between *TCF7* and pro-caspase-8, genetic knockdown and rescue experiments were performed in Jurkat T cells. Short hairpin RNA targeting human *TCF7* (shRNA) and a scramble control shRNA were purchased from OriGene with Cat.No.TR30004. For rescue experiments, a full-length human *TCF7* cDNA clone (OriGene, Cat. No. RC224338) was utilized. The Jurkat T cells were transduced with lentiviral particles or transiently transfected with shControl or sh*TCF7*. After 48 h, targeted cells were further co-transfected with the *TCF7* rescue plasmid or empty vector using a suspension cell transfection reagent. After 48 h of dual-transfection, cells were harvested for Western blot analysis.

### 4.18. Statistical Analyses

All statistical analyses were conducted in RStudio (version 4.5.1). Continuous variables were analyzed using the Wilcoxon rank-sum test (two-tailed) unless otherwise specified. Correlation analyses were conducted using Spearman’s rank correlation coefficient. Receiver operating characteristic (ROC) curves, calibration curves, and decision curve analysis (DCA) were employed to evaluate diagnostic performance and clinical utility. To handle categories with low expected frequencies, a Monte Carlo simulation (B = 2000 replicates) was applied to ensure robust *p*-value estimation. For in vitro and ex vivo quantitative experiments, statistical significance between experimental groups was assessed using a two-tailed Student’s *t*-test. Crucially, Welch’s correction was strictly applied to the *t*-test when variances between groups were unequal, ensuring robust statistical estimation. All the statistical tests were two-sided, and *p* < 0.05 was considered significant (* *p* < 0.05, ** *p* < 0.01, *** *p* < 0.001).

## 5. Conclusions

In this study, we integrated large-scale blood transcriptomic data with machine learning, single-cell analysis, human tissue immunofluorescence, and ex vivo/in vitro functional experiments to identify a three-gene signature (*RORC*, *TCF7*, and *CLEC4D*) associated with systemic immune dysregulation in COPD. The signature showed consistent diagnostic performance across multiple independent cohorts and tissues. Mechanistic investigations, including experiments in patient-derived cells and genetic rescue models, suggested that *TCF7* positively regulates pro-caspase-8 expression in T lymphocytes, and that this apoptotic-related axis is disrupted in the COPD pulmonary microenvironment. Furthermore, pharmacological validation indicated that Celecoxib and Lovastatin exert anti-inflammatory effects that may be partially mediated through the preservation of *RORC*. While these findings provide a preliminary framework for understanding COPD immune dysregulation and identifying potential repurposable drug candidates, direct in vivo causality remains to be established. Future studies using animal models and prospective clinical cohorts are warranted to further evaluate the translational potential of this molecular signature.

## Figures and Tables

**Table 1:** Baseline characteristics of non-statin patients (COPD vs. Control).

Variable	COPD (*n* = 405)	Control (*n* = 44)	*p*-Value
Sample Size, *n*	405	44	
Age (years), Mean ± SD	63.4 ± 6.4	58.6 ± 6.2	<0.001
Sex, *n* (%)			1.000
F	149 (36.8%)	16 (36.4%)	
M	256 (63.2%)	28 (63.6%)	
Smoking Status, *n* (%)			<0.001
Current smoker	91 (22.5%)	0 (0.0%)	
Former smoker	314 (77.5%)	22 (50.0%)	
Never smoked	0 (0.0%)	22 (50.0%)	
FEV1% Predicted, Mean ± SD	49.8 ± 16.0	109.5 ± 16.5	<0.001
FEV1/FVC, Mean ± SD	45.0 ± 11.9	79.0 ± 6.1	<0.001

Note: Continuous variables presented as mean ± SD. *p*-values from Wilcoxon rank-sum test (continuous) or Fisher’s exact test with Monte Carlo simulation (categorical).

**Table 2:** Intersected drugs of cMAP and DsigDB with FDR < 0.05 while CS < 0 targeting the *RORC* gene. Among these drugs, Lovastatin and Celecoxib were prioritized for subsequent molecular docking with *RORC* based on their therapeutic relevance and established mechanisms of action.

Drug	MOA	CS Score
Progesterone	Progesterone receptor agonist	−1.4303
Rofecoxib	Cyclooxygenase inhibitor	−1.2957
Nifedipine	Calcium channel blocker	−1.2368
Mitotane	Carcinogen	−1.1911
Lovastatin	HMGCR inhibitor	−1.1305
Liothyronine	Thyroid hormone stimulant	−1.1246
Digoxin	Na/K-ATPase inhibitor	−1.0575
Celecoxib	Cyclooxygenase inhibitor	−0.997
Colchicine	Tubulin inhibitor	−0.9446
Estradiol	Estrogen receptor agonist	−0.8209

**Table 3:** Clinical information of patients enrolled.

Variable	COPD (N = 15)	Control (N = 15)	*p*-Value
Age (years), Mean ± SD	66.8 ± 5.2	64.1 ± 5.0	0.177
Male, *n* (%)	12 (80.0%)	10 (66.7%)	0.682
BMI (kg/m^2^), Mean ± SD	21.6 ± 2.3	23.5 ± 1.5	0.02
Smoking Status, *n* (%)			0.01
Never smoker	0 (0.0%)	7 (46.7%)	
Former smoker	9 (60.0%)	6 (40.0%)	
Current smoker	6 (40.0%)	2 (13.3%)	
Pack-years, Median [IQR]	45.0 [32.5, 55.0]	10.0 [0.0, 22.5]	<0.001
FEV1/FVC (%), Mean ± SD	57.6 ± 8.2	78.2 ± 3.8	<0.001
FEV1% Predicted, Median [IQR]	60.0 [48.5, 69.0]	93.0 [89.0, 99.0]	<0.001
GOLD Stage, *n* (%)			<0.001
Stage 1	2 (13.3%)	0 (0.0%)	
Stage 2	9 (60.0%)	0 (0.0%)	
Stage 3	4 (26.7%)	0 (0.0%)	
Blood Neutrophils (×10^9^/L), Median [IQR]	4.90 [4.40, 5.40]	3.20 [2.95, 3.70]	<0.001
Blood Eosinophils (×10^9^/L), Median [IQR]	0.14 [0.11, 0.17]	0.15 [0.12, 0.18]	0.589

Note: Continuous variables presented as mean ± SD or median [IQR]. *p*-values from Wilcoxon rank−sum test (continuous) or Fisher’s exact test with Monte Carlo simulation (categorical).

**Table 4:** Specific information on GSE cohorts.

Dataset ID	Platform	Cohort Type	Sample Source	Sample Size (COPD/Control)	Age (Mean ± SD)	Male (%)	Smoking Status		Authors (Reference)
							COPD	Control	
GSE71220	GPL11532	Training set	Blood	405/44	63.0 ± 6.5	63.3	91 Current,314 Former	22 Former,22 Never	McManus et al. [45]
GSE42057	GPL570	Testing set	Blood	94/42	63.1 ± 8.6	54.4	22 Current,72 Former	13 Current,23 Former,6 Unknown	Hughes et al. [46]
GSE24709	GPL9040	miRNA set	Blood	24/19	Unknown	Unknown	Unknown	Unknown	Kellar et al. [47]
GSE249584	GPL24676	Single-cell set	Blood	8/7	Unknown	73.3	Unknown	Unknown	Heo et al. [48]
GSE56766	GPL570	External Validation 1	Blood	49/29	Unknown	55.1	Unknown	24 Current,5 Never	Roberts et al. [49]
GSE306950	GPL30209	External Validation 2	Blood	10/25	Unknown	8.6	Unknown	Unknown	Qiao et al. [50]
GSE106986	GPL13497	External Validation 3	Lung	5/14	66.8 ± 9.8	63.2	6 Current,8 Former	5 Never	Marwitz et al. [51]

## Data Availability

The datasets analyzed in this study are publicly available in the Gene Expression Omnibus (GEO) repository (https://www.ncbi.nlm.nih.gov/geo/) under the accession numbers GSE71220, GSE42057, GSE106986, GSE56766, GSE306950, and GSE249584.

## Supplementary Materials

The following supporting information can be downloaded at: https://www.mdpi.com/article/10.3390/ijms27104231/s1.
