# Supplementary figures and images for: Unfolding Immune Dysregulation in COPD: Identification of a Three-Gene Signature and Functional Validation of TCF7 in Human Lung Tissue and T Lymphocytes
Source: Int J Mol Sci. 2026 May 9;27(10):4231. doi: 10.3390/ijms27104231 (PMC13206788; doi:10.3390/ijms27104231)

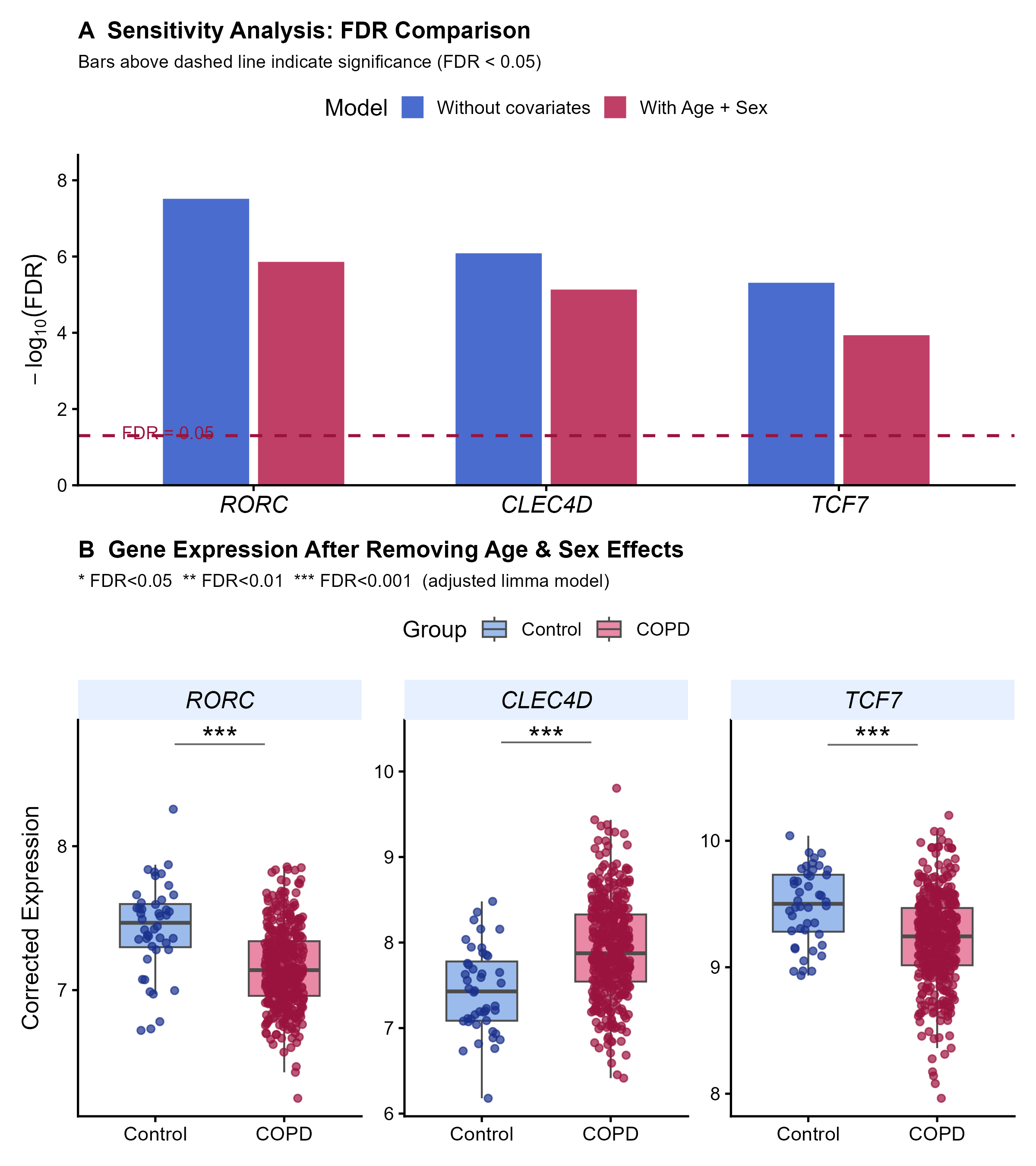

Supplement: Supplementary file 1 [file ijms-27-04231-s001.zip › Supplementary Figure S1.png]

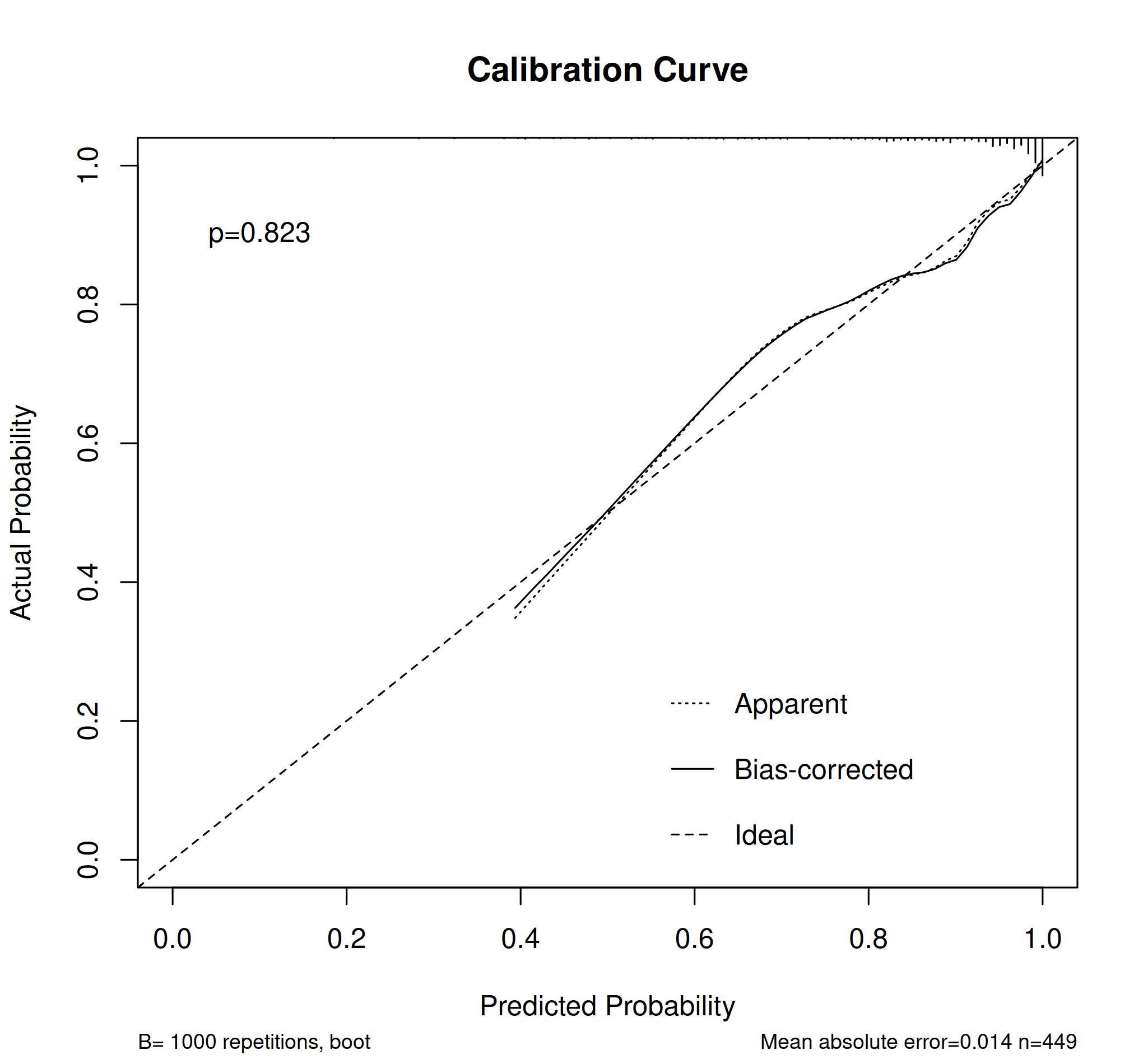

Supplement: Supplementary file 1 [file ijms-27-04231-s001.zip › Supplementary Figure S2.png]

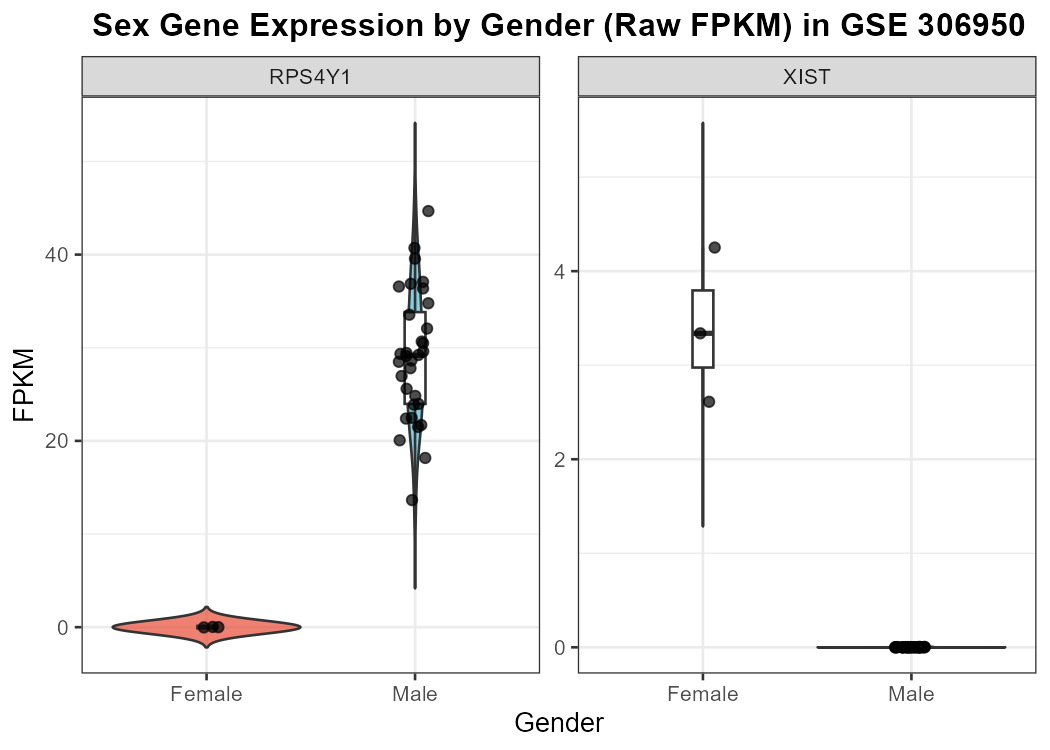

Supplement: Supplementary file 1 [file ijms-27-04231-s001.zip › Supplementary Figure S3.png]

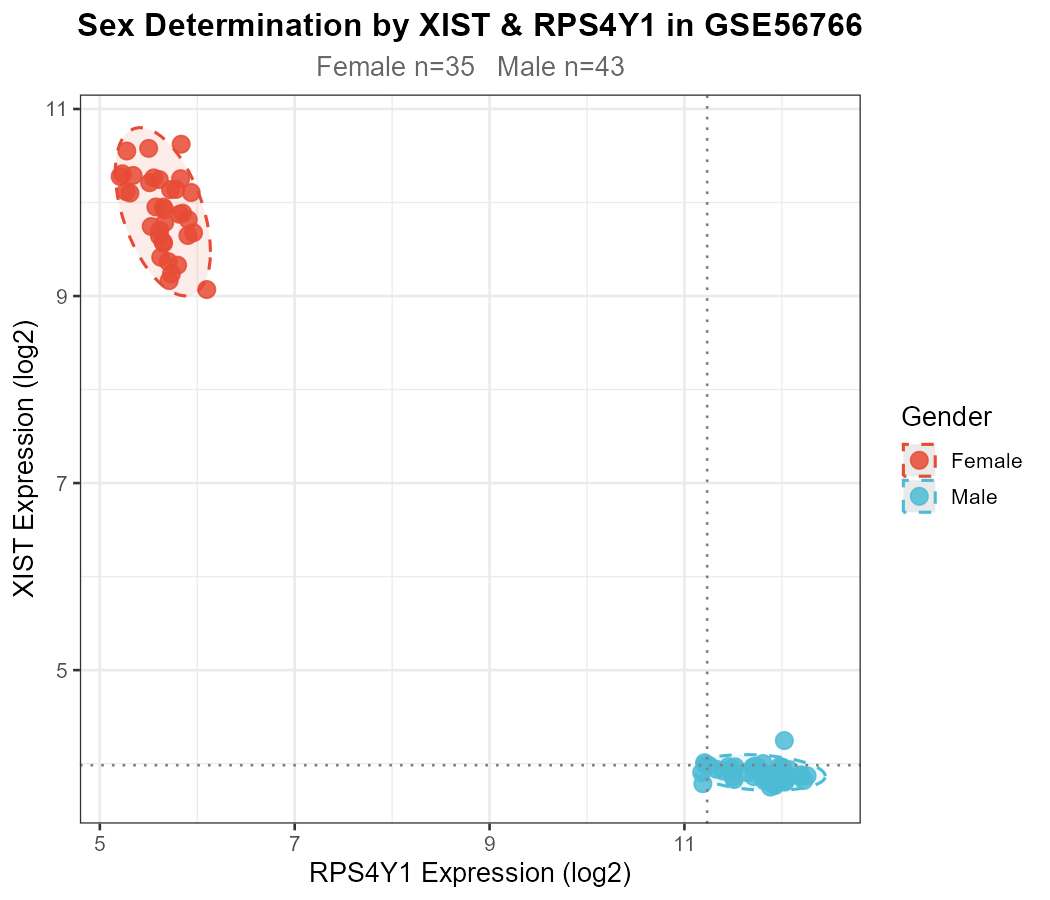

Supplement: Supplementary file 1 [file ijms-27-04231-s001.zip › Supplementary Figure S4.png]

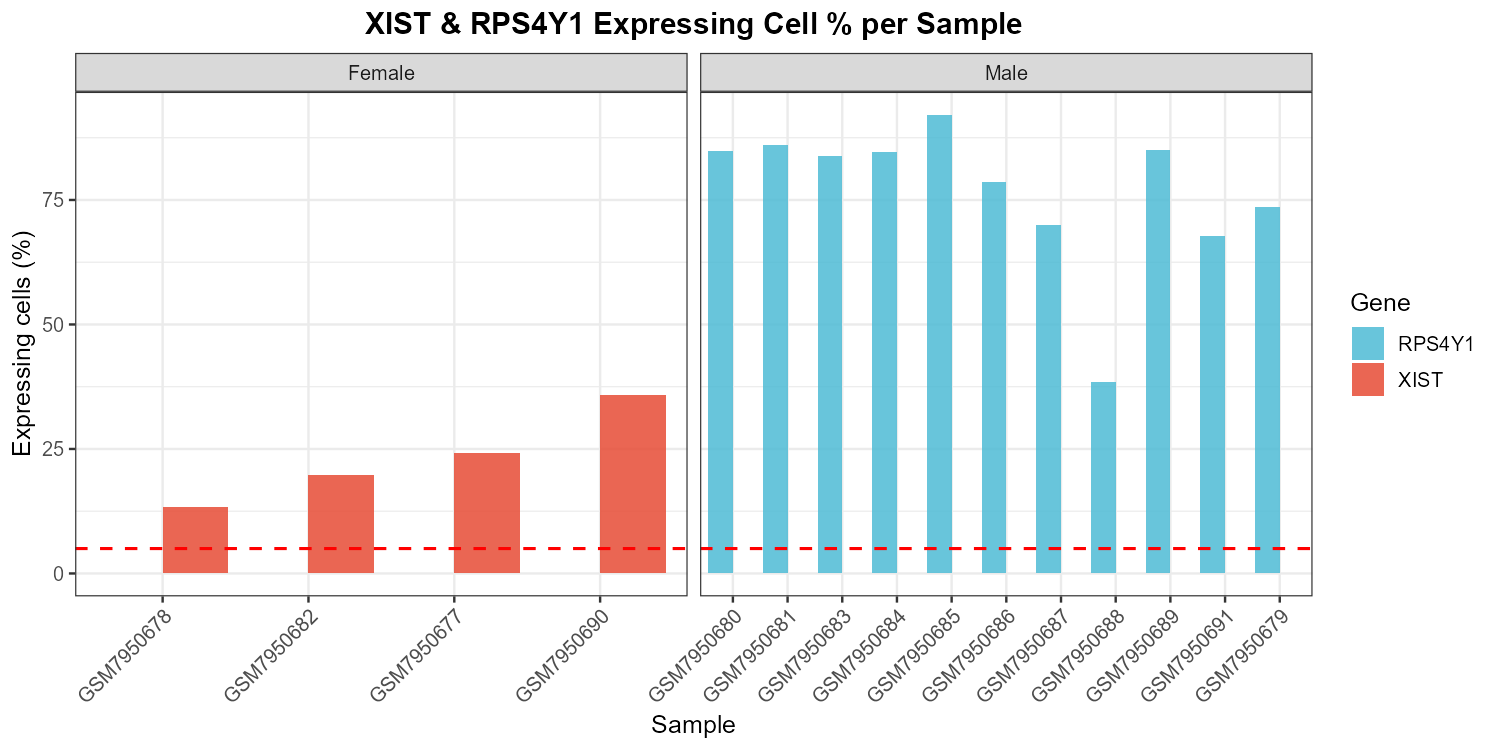

Supplement: Supplementary file 1 [file ijms-27-04231-s001.zip › Supplementary Figure S5.png]
